# LADEC: The Large Database of English Compounds

**DOI:** 10.3758/s13428-019-01282-6

**Published:** 2019-07-25

**Authors:** Christina L. Gagné, Thomas L. Spalding, Daniel Schmidtke

**Affiliations:** 1 Department of Psychology, University of Alberta, Edmonton, Alberta Canada; 2 Department of Linguistics and Languages, McMaster University, Hamilton, Ontario Canada

**Keywords:** Compound words, Semantic transparency, Psycholinguistics, Morphology, Bigram frequency, Sentiment, Family size

## Abstract

The Large Database of English Compounds (LADEC) consists of over 8,000 English words that can be parsed into two constituents that are free morphemes, making it the largest existing database specifically for use in research on compound words. Both monomorphemic (e.g., *wheel*) and multimorphemic (e.g., *teacher*) constituents were used. The items were selected from a range of sources, including CELEX, the English Lexicon Project, the British Lexicon Project, the British National Corpus, and Wordnet, and were hand-coded as compounds (e.g., *snowball*). Participants rated each compound in terms of how predictable its meaning is from its parts, as well as the extent to which each constituent retains its meaning in the compound. In addition, we obtained linguistic characteristics that might influence compound processing (e.g., frequency, family size, and bigram frequency). To show the usefulness of the database in investigating compound processing, we conducted a number of analyses that showed that compound processing is consistently affected by semantic transparency, as well as by many of the other variables included in LADEC. We also showed that the effects of the variables associated with the two constituents are not symmetric. In short, LADEC provides the opportunity for researchers to investigate a number of questions about compounds that have not been possible to investigate in the past, due to the lack of sufficiently large and robust datasets. In addition to directly allowing researchers to test hypotheses using the information included in LADEC, the database will contribute to future compound research by allowing better stimulus selection and matching.

Linguistic and psycholinguistic research has benefited greatly from the use of large-scale databases from which researchers can select stimuli for their work, as well as look up characteristics of existing materials. For example, the CELEX database (Baayen, Piepenbrock, & Gulikers, 1995), SUBLEX-US (Brysbaert & New, 2009), and SUBLEX-UK (van Heuven, Mandera, Keuleers, & Brysbaert, 2014) have been widely used for determining word-frequency and the British Lexicon Project (BLP: Keuleers, Lacey, Rastle, & Brysbaert, 2012) and the English Lexicon Project (ELP: Balota et al., 2007) provide information about behavioural performance measures (lexical decision latencies and naming times). In terms of other variables, for example, Brysbaert, Warriner, and Kuperman (2014) provide concreteness norms, whereas Warriner, Kuperman, and Brysbaert (2013) provide valence ratings. These databases have provided valuable insight into the processing of mono- and multimorphemic words. However, they are not ideally suited for research involving compound words. First, compound words cannot be readily identified and extracted; these databases contain multimorphemic words, but do not identify compounds separately. Thus, a search in the ELP for bimorphemic items returns items such as *bulls*, *delays*, and *vindicate* in addition to compounds. The BLP identifies complex words, but this category includes words such as *blazer* and *boorish* in addition to compounds. Second, the list of compounds is incomplete. The BLP database, for example, includes only monosyllabic and bisyllabic words, and consequently, multisyllabic compounds (e.g., *thunderstorm*, *schoolteacher*, and *bumblebee*) are excluded. Third, the databases do not include measures, such as semantic transparency, that are particularly relevant to compounds.

The aim of the present project was to provide a large-scale database of English closed compounds (also called concatenated compounds) along with a range of their orthographic, morphological, and semantic properties that are relevant for psycholinguistic, corpus, neurolinguistic and computational linguistic research. These properties include various measures of semantic transparency (based on human ratings and measures of association), family size, and bigram frequency at the morpheme boundary. The database fills an important gap in the field because it includes measures that are unique to compound words but missing from existing datasets.

Two other databases, Juhasz, Lai, and Woodcock’s (2015) and the recently published Kim, Yap, and Goh (2018), also contain compounds and transparency ratings. However, our database extends rather than duplicates these databases, and thus provides a useful additional resource. For example, these previous databases contain items from ELP, whereas for ours we recruited compounds from ELP but also from additional sources, which yielded a much larger pool of compounds. Also, our items were hand-coded, and thus we were able to capture items that had not previously been identified as compounds. We also included a variety of other measures that are useful for compound research, such as bigram frequency and family size.

In terms of the ratings per se, the present set of ratings and the existing ratings differ in terms of the nature of their questions. First, in terms of types of ratings, Juhasz et al. (2015) obtained one rating pertaining to the entire compound, whereas Kim et al. (2018) obtained constituent-based ratings, one per each constituent. In the present project, we collected both a compound measure of semantic transparency (similar to that in Juhasz et al., 2015) and constituent measures (similar to those in Kim et al., 2018) from the same population.

Second, the type of information targeted by the questions used to obtain the human ratings differed. Our ratings targeted semantic transparency, by directly asking participants about meaning retention (e.g., how much of the meaning of *pillow* is retained in *pillowcase*) and meaning predictability (e.g., how predictable is the meaning of *pillowcase* from the meanings of *pillow* and *case*). Juhasz et al. (2015) asked participants to indicate whether the two constituents were “transparently related to the meaning of the compound.” Kim et al. (2018) asked participants to indicate how strongly related a constituent and the compound were (e.g., *snow*–*snowball*). Relatedness measures are useful but are not specific to semantic transparency (e.g., *dog* and *bone* are related, as are *cold* and *hot*). Therefore, the three databases differ in terms of the types and natures of their ratings. It is useful to have sets of ratings that target different aspects of the relationship between a compound and its morphological constituents.

Another advantage of the new dataset is that it contains a larger set of compounds than existing databases do. Having a large set of compounds is useful for selecting materials for experiments in that it allows greater flexibility in terms of matching items on various characteristics. Also, having a larger set of items yields more precise measure of statistics (e.g., mean, standard deviation, and range) associated with the distribution of relevant variables such as semantic transparency. Furthermore, the larger set can yield a more representative picture of how various variables are related to each other. For example, it can be used to examine the association between response time and semantic transparency. Having a large set of human ratings of semantic transparency can also be useful for comparing and testing against computational-based measures, because human measures and distributional-semantics-based measures, for example, do not always capture the same information (Gagné, Spalding, & Nisbet, 2016).

In this article, we begin by providing a brief overview of why research on morphologically complex words is useful. Then we provide details about the database project, by first explaining how the compounds were selected and then providing details about how we obtained various linguistic and psycholinguistic variables pertaining to the compounds. In the latter part of the article, we use the database to test several theoretically based hypotheses, as a demonstration of the usefulness of the new database.

## The importance of understanding the processing of compound words

The intended meanings of words can often be inferred from a word’s morphemic structure. In fact, approximately half of the new words that students encounter in books have meanings that can be derived via morphemic analysis; Nagy and Anderson (1984) and Nagy, Anderson, Schommer, Scott, and Stallman (1989) have reported that more than 60% of the new words that readers encounter contain morphological structures that allow the reader to make a reasonable guess about the meaning of the whole word. Indeed, readers often use word parts to determine word meaning (Graves, 2006), and the awareness of morphemic structure has been linked with enhanced vocabulary growth (Baumann et al., 2002; Brusnighan & Folk, 2012; Levin, Carney, & Pressley, 1988) and reading comprehension outcomes, in both developing readers (McCutchen, Logan, & Biangardi-Orpe, 2009; Nagy, Carlisle, & Goodwin, 2013) and university students (Kemp & Bryant, 2003).

Precisely how the language system makes use of morphological structure is not yet fully understood, and a key empirical and theoretical question has centered on the extent to which representations of the constituents are accessed during the processing of multimorphemic words. For example, what role do *sea* and *bird* play in the representation and processing of the compound word *seabird*? There is much debate among linguists and psycholinguists about how morphological information is represented in the mental lexicon: Are complex words stored as full forms, such that morphological structure plays no (or a minimal) role (Butterworth, 1983; Lukatela, Carello, & Turvey, 1987; Manelis & Tharp, 1977), or as combinations of their constituent morphemes (Chialant & Caramazza, 1995; Dell, 1986; Frauenfelder & Schreuder, 1991; Laudanna & Burani, 1995; Schreuder & Baayen, 1995, 1997; Taft & Forster, 1975, 1976)? The latter theories vary in terms of the point at which the constituents’ representations become available (see Kuperman, Bertram, & Baayen, 2010, for an overview). Many articles in the literature on complex-word processing have been explicitly concerned with establishing how the balance of lexical storage and morphologically based computation affects lexical processing (e.g., Bertram, Schreuder, & Baayen, 2000; Butterworth, 1983; Bybee, 1995; Kuperman, Bertram, & Baayen, 2010; Libben, 2005).

Compound words (e.g., *snowball*, *bluebird*) offer an ideal test case for addressing this ongoing debate because the constituents of compounds are themselves words that have their own lexical and semantic representations. Consequently, “compound words present a much greater challenge to online morphological parsing than do affixed words” (Libben, 2005, p. 270). For example, because the constituents of compounds are an open-class set, there is no reliable heuristic for parsing compounds (as compared with words containing affixes such as -*ed*, -*s*, and -*ing*, which are a closed-class set, so that the words can be parsed by removing the affixes from the stem). Recent psycholinguistic theories have claimed that the language system activates as many representations as possible during the comprehension of compounds, including the representations of the compounds’ constituents, such as *neck* and *lace* for *necklace* (Ji, Gagné, & Spalding, 2011; Libben, 2005, 2010), and that constituent integration involves actively constructing a meaning by integrating the semantic representations of the constituents (Fiorentino & Poeppel, 2007; Gagné & Spalding, 2009; Gagné, Spalding, Figueredo, & Mullaly, 2009; Ji et al., 2011).

Schmidtke and Kuperman (2019) recently showed that many orthographic, morphological, and semantic sources of information pertaining to compound words and their morphemes become available concurrently and very quickly (within a maximum time window of about 260 ms after reading a compound word). Consistent with this notion, considerable evidence has shown that the lexical representations of word constituents are activated during word processing (Andrews, 1986; Juhasz, Starr, Inhoff, & Placke, 2003; Libben, 1998; Pollatsek, Hyönä, & Bertram, 2000). There has also been some indication of a morphological effect that is distinct from effects such as the semantic effect and phonological effect (e.g., Assink & Sandra, 2003; Bentin & Feldman, 1990; Frost, Kugler, Deutsch, & Forster, 2005; Gumnior, Bölte, & Zwitserlood, 2006). Clearly, research on compound words plays a central role in investigating these issues. Thus, a large database of compound words and their associated information, such as LADEC, can have an immediate and large impact on an important and central area of psycholinguistic research. Theoretical issues about the nature of complex-word processing and about the representation of compound words can be aided by the availability of a larger pool of compounds that represent the entire range of the distribution of the lexical variable under question.

## Creation of a set of English compounds

Our goal was to obtain a set of two-constituent English compounds using a clearly defined algorithm that would produce as comprehensive a set as possible, given the existing input materials. Our focus was on concatenated compounds (i.e., unspaced compounds), because this type of compound is particularly challenging to extract from existing databases and text unless they have previously been coded as compounds.

For reasons noted above, a comprehensive list could not be obtained via a direct search and aggregation of existing linguistic databases. Thus, we developed a procedure in which we first identified potential constituents, then created a set of all possible combinations of those constituents, and finally identified which of those combinations are existing English words. Both monomorphemic (e.g., *wheel*) and multimorphemic (e.g., *teacher*) constituents were used. In this section, we discuss each of these steps.

### Obtaining potential constituents

The potential constituents, referred to here as “bases,” were obtained from four primary sources: the set of items contained in Brysbaert et al. (2014), all nouns and adjectives in the English Lexicon Project (Balota et al., 2007), Mathematica’s Word Dictionary, and Mathematica’s WordData set. Mathematica’s WordData includes materials from a range of sources, including the British National Corpus and WordNet. We opted to restrict the bases in terms of word length such that they were 3–10 letters long, because this would produce compounds that ranged from 6 to 20 letters long. These sources yielded 76,424 bases. Some bases, such as *hood*, *ship*, *ion*, and *ness*, also can function as suffixes, but they were maintained in the set for completeness because they have a noun sense (e.g., *ion* is type of atom or molecule, and *ness* is a headland) as well as noun classifications in Corpus of Contemporary American English (COCA) and Wordnet. That is, no bases were excluded. In the next step of the project (see the following subsection: Creating Word–Word Items), we identified items that were compounds (e.g., *spaceship* and *monkshood*) and those that were not (e.g., *friendship* and *motherhood*). Table 1 provides the frequency distributions of the letter lengths for the bases. In terms of the number of morphemes, 50,615 of the bases were in the ELP database. Of these, 19% were classified as being monomorphemic.

**Table 1:** Frequency distribution of letter lengths for the bases

Length	Frequency
3	795
4	3,000
5	6,077
6	10,049
7	13,963
8	15,618
9	14,734
10	12,180

#### Creating word–word items

The bases were combined pairwise to produce a list of all possible word–word combinations, which yielded over 5.8 billion unique items. We then identified items on this list that were existing English words. We did this by first creating a set of existing words by creating a unique list of items from all nouns in WordNet, all items (excluding proper names and abbreviations) in Mathematica’s dictionary of English words, all items in the English Lexicon Project (Balota et al., 2007), and all items in Brysbaert et al. (2014). We extracted 28,360 word–word combinations that appeared in this list of existing English words. Because our aim was to obtain compounds that could be parsed into two words, compounds that contained reductions (e.g., pastime or chaffinch) were not obtained via our procedure.

We further restricted this list to items that appeared as nouns in Wordnet, which resulted in a final set of 16,697 word–word items. The remaining 11,663 non-noun items (e.g., *seasick* is an adjective) were excluded from further analysis. Our focus on nouns was intended to produce a homogeneous set of items overall and a manageable set of items for the subsequent hand-coding stage and for rating experiments.

#### Identifying compounds

The set of 16,697 word–word items contained both compounds (e.g., *necklace*) as well as pseudo-compounds (e.g., *carpet* contains the English morphemes *car* and *pet*, but they do not function as morphemes in *carpet*). Therefore, our next step was to identify the genuine compound words. We began by comparing the list to a set of known compounds. The set of known compounds was an aggregated list based on items that were coded as either adjective–noun or noun–noun in the Brown corpus, CELEX corpus, or COCA, as well as the set of phrases in Costello, Veale, and Dunne (2006). Searches in CELEX for NN and AN items yielded both open (e.g., *American football*) and closed (e.g., *afterglow* and *backyard*) compounds, as well as noncompounds (e.g., *Puritanism*, *Oxbridge*, and *tarmac*). All open or hyphenated compounds were converted to closed compounds for the purpose of identifying known compounds among our list. This initial step yielded 2,578 compounds among our list of 16,697 word–word items. The remaining 14,351 items were manually coded (over a 3-year period) by trained research assistants as to whether they were compounds. Each item was coded by at least two people. The *Oxford English Dictionary* was consulted for words that were unfamiliar to the coders, or for items for which there was uncertainty as to their etymologic history. Any disagreements in coding were resolved via discussion.

In total, we obtained a final set of 8,956 items. This number includes all instances of items with multiple parses; for example, *bluestone* can be divided into *blue + stone* or *blues + tone* or *bluest + one*, and thus it has three entries in the database, one for each of these parses. The vast majority of compounds (7,804) had only one unique parse, 564 had two parses, and eight had three parses, for a total of 8,376 unique compounds. We kept the incorrect parses in the database in accordance  with our aim of having an inclusive database. This feature is novel, in that it is not part of other compound databases. It provides a useful resource for researchers who want to select stimuli that have only one word–word parse.

This set of compounds is, to our knowledge, the largest set of identified English compounds in a psycholinguistic database. For comparison, Kim, Yap, and Goh (2018) contains 2,861 compounds, Juhasz et al.’s (2015) list contains 629 compounds, and Libben (2005) notes that the English CELEX database contains 1,437 noun–noun compounds. Our set also is larger than those found in BLP and ELP. Importantly, our algorithm and subsequent coding identified 4,605 compounds that were not present in either the ELP or BLP corpora, and thus extends the number of compounds available to researchers. Furthermore, our list of items indicates that ELP and BLP contain largely nonoverlapping sets of compounds. Only 1,587 items are found in both corpora; 1,365 are contained in ELP but not in BLP, and 824 are found in BLP but not in ELP.

## Obtaining psycholinguistic and linguistic variables

Our goal for LADEC was to provide researchers with a large set of compounds, along with associated information that will be most useful in the psycholinguistic study of compound word processing. Thus, after obtaining the set of compounds, we obtained information about a variety of psycholinguistic and linguistic characteristics for the compounds and constituents. In addition to various measures of semantic transparency, which is our primary focus in this database, we included variables that have been associated in previous research (de Jong, Feldman, Schreuder, Pastizzo, & Baayen, 2002; de Jong, Schreuder, & Baayen, 2000; Gagné & Spalding, 2009; Kuperman et al., 2010; Leminen, Lehtonen, Bozic, & Clahsen, 2016; Schmidtke & Kuperman, 2019) with processing costs of compound words in language comprehension across a range of behavioral (primed and unprimed visual lexical decision, eye tracking, visual-world paradigm) and neurophysiological (electro-encephalography and magneto-encephalography) studies, and also in experiments of language production (typing, naming, free recall), as well as other variables that would be useful for researchers using the database to select materials.

### Semantic transparency measures

Semantic transparency reflects the relationship between the compound and its morphemic constituents. For example, the constituents *blue* and *berry* both appear to play a role in the meaning of *blueberry* whereas the constituents *honey* and *moon* seem unrelated to the meaning of *honeymoon*. The notion of semantic transparency has played a central role in testing competing theories of linguistic representation as researchers have sought to examine how semantic transparency influences the segmentation of morphologically complex words as well as how lexical representations of constituents might be connected (or not) to the semantic representation of the whole compound (Fiorentino & Fund-Reznicek, 2009; Gagné & Spalding, 2009; Ji et al., 2011; Marelli & Luzzatti, 2012; Monsell, 1985; Sandra, 1990). Schmidtke, Van Dyke, and Kuperman (2018) have reported main effects of left-whole transparency (i.e., the transparency associated with the first constituent and the whole compound) across a whole swath of eye-movement measures during naturalistic reading, and interactions between language experience and right-whole transparency (i.e., the transparency associated with the second constituent and the whole compound) in later word reading measures (e.g., in total reading time).

Despite the prominence of semantic transparency in compound research, there has not yet been common agreement about how best to define and measure this construct. Semantic transparency has been described in terms of the degree to which the meaning of the constituent is retained in the meaning of the whole compound, and also in terms of the degree to which the meaning of the compound is predictable from the meaning of the constituents. In sum, the construct has been discussed both in the context of the whole compound as well as in the context of the individual constituents. These approaches to defining semantic transparency reflect different facets of this construct. For example, measures of meaning retention appear to reflect the semantic similarity between a compound’s meaning and the constituent meaning, whereas measures of predictability might indicate the degree of semantic compositionality of the compound’s concept (see Marelli & Luzzatti, 2012). Another way that semantic transparency has been defined is in terms of the relatedness/association between the constituents and the compound (Kuperman, 2013; Wang, Hsu, Tien, & Pomplun, 2014). In addition to differences in whether measures of transparency are constituent-based or compound-based, semantic transparency has been operationalized in various ways, ranging from dichotomous classification by the researcher to participant ratings to measures derived via distributional semantics (e.g., Gunther & Marelli, 2018; Landauer, 2002; Mandera, Keuleers, & Brysbaert, 2017). The various measures do not necessarily reflect the same underlying aspects of semantic transparency (Gagné et al., 2016).

For our project, we included several variables that reflect semantic transparency, to give researchers a range of options. Having transparency measures for a large set of compounds will allow researchers to further test whether and how semantic transparency is involved in the processing of complex words. To date, the only existing sets of ratings have been presented in Juhasz et al. (2015) for approximately 600 compounds, and more recently in Schmidtke, Van Dyke, and Kuperman (2018) for 455 compounds, and Kim et al. (2018) for 2,861 compounds. Having semantic transparency information for over 8,000 compounds would greatly expand the possibilities for further research. In addition, our database contains a compound-based rating, as well as constituent-based ratings.

### Semantic transparency ratings

We used a rating task to gather meaning retention and meaning predictability judgments for 8,515 items; 8,304 of these were compounds in the database, and the remaining items were pseudo-compounds (i.e., *mushroom* and *booklet*). Some pseudo-compounds were included in the rating study because they had been incorrectly tagged in other databases or articles as being compounds and had been marked as “known compounds” in our initial classification prior to our completion of hand-coding these items. The ratings were obtained in a series of seven experiments, each involving a unique set of participants. Within each experiment, we obtained three measures of semantic transparency: one for each constituent, and one for the entire compound. The two ratings that specifically targeted the transparency of the individual constituents (e.g., *snow* and *ball* in the context of the compound *snowball*) allowed us to distinguish between the three types of opaque compounds: fully opaque compounds (e.g., *dumbbell* and *honeymoon*), compounds with opaque heads (e.g., *flowerbed* and *bookworm*), and compounds with opaque modifiers (e.g., *raspberry* and *chestnut*). The compound-based rating provides an overall measure of how transparent the compound is as a whole.

#### Materials and participants

The compounds were randomly divided into lists ranging from 118 to 150 items. In all, 1,772 native speakers of English from the University of Alberta participated in the study and received partial course credit for their participation. Each participant was tested individually in the laboratory and each received one list. The order of presentation was uniquely randomized for each participant.

#### Procedure

In Part 1 of the experiment, participants viewed each compound on a computer screen and rated how predictable its meaning is from its parts (e.g., “How predictable is the meaning of *flowerbed* from the meaning of *flower* and *bed*?”). To make their rating, participants used the mouse to position a slider bar labeled with the endpoints “very predictable” and “not very predictable” (the same labels as used in previous work, including Libben, Gibson, Yoon, & Sandra, 2003). In Part 2 of the experiment, participants rated the extent that each constituent retains its meaning in the compound (e.g., “How much does *flower* retain its meaning in flowerbed?” and “How much does *bed* retain its meaning in *flowerbed?*”). The endpoints of the scale were labeled “retains none of its meaning” and “retains all of its meaning.” The default start position for the slider was 50%. The set of items included both correct parses (e.g., *grave* + *stone*) and incorrect parses (e.g., *graves* + *tone*), but each participant saw only one version.

#### Results

Prior to data analysis, we removed participants who submitted 50% (the default position) as a response on more than 100 items. The data from participants who did not finish the task were also removed. The ratings were aggregated in order to obtain the mean rating for each item. Each item was rated by 21 to 44 people. For each experimental list, we conducted split-half interrater reliability assessments for each transparency measure. Split-half reliability was high across all lists for each transparency measure, and ranged from .83 to .93: ratings for the first constituent (C1: List 1, *r* = .880; List 2, *r* = .874; List 3, *r* = .905; List 4, *r* = .881; List 5, *r* = .877; List 6, *r* = .909; List 7, *r* = .934); ratings for the second constituent (C2: List 1, *r* = .826; List 2, *r* = .843; List 3, *r* = .874; List 4, *r* = .840; List 5, *r* = .842; List 6, *r* = .944; List 7, *r* = .924), and ratings for the whole compound (List 1, *r* = .898; List 2, *r* = .881; List 3, *r* = .896; List 4, *r* = .888; List 5, *r* = .838; List 6, *r* = .907; List 7, *r* = .912). For comparison, the reliability ratings for Kim et al. (2018) were .78 for the first-constituent ratings and .77 for the second-constituent ratings.

**Fig. 1:**
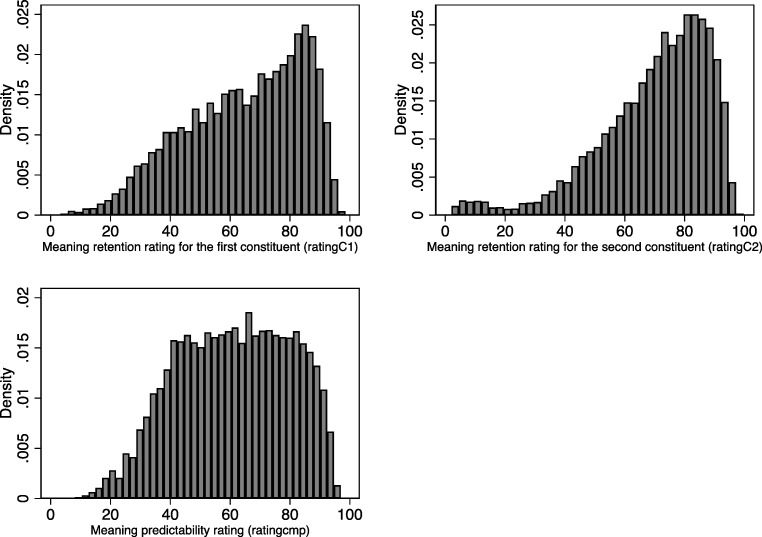
Histograms of semantic transparency ratings for the first constituent (ratingC1, top left), second constituent (ratingC2, top right), and full compound (ratingcmp, bottom)

Descriptive statistics for the meaning predictability rating and the two measures of meaning retention (one for each constituent) are provided, for all items and separately for the correctly parsed and incorrectly parsed items, in Table 2. Histograms of each of the three ratings are provided in Fig. 1.

**Table 2.** Descriptive statistics for semantic transparency ratings for the first constituent (ratingC1), second constituent (ratingC2), and full compound (ratingcmp), reported separately for all items, for correctly parsed items only, and for incorrectly parsed items only

	*N*	Mean	*SD*	Min	Max
All Items					
ratingC1	8,299	64.59	(19.68)	3.28	98.54
ratingC2	8,299	69.70	(18.55)	2.04	99.68
ratingcmp	8,299	61.00	(18.80)	8.14	96.96
Correctly Parsed Items					
ratingC1	8,115	64.80	(19.59)	4.90	98.54
ratingC2	8,115	71.00	(16.46)	3.13	99.68
ratingcmp	8,115	61.90	(18.01)	14.37	96.96
Incorrectly Parsed Items					
ratingC1	184	55.73	(21.92)	3.28	94.32
ratingC2	184	11.78	(11.64)	2.04	85.03
ratingcmp	184	21.84	(7.43)	8.14	61.90

The items in the database represent a wide range of semantic transparencies, and this aspect of the database will be useful for selecting stimuli for experiments. Items at the higher end of the meaning predictability ratings for correctly parsed compounds include *schoolyard*, *seashell*, *anklebone*, *bookstore*, and *woodcarvings.* Items at the lower end of the meaning predictability ratings include *butterfly*, *magpie*, *dumbbell*, *tomboy*, and *hamstring.* For the meaning retention ratings for the first constituent, items with higher ratings include *waterfall*, *toothache*, *and hilltop*, and items with lower ratings include *witchgrass*, *turtleneck*, and *peacoat*. For the meaning retention ratings for the second constituent, items with higher ratings include *woodpile*, *windstorm*, *cellphone*, *butterflyfish*, and *candlelight*, and items with lower ratings include *stairwell*, *bookworm*, *brainstorm*, *copycat*, and *hotdog.*

In terms of the incorrectly parsed items, it might seem surprising that some of these items had ratings at the upper end of the scale. However, we inspected the cases in which the ratings were greater than 80%, and in those cases, the incorrect first constituent was still semantically related to the compound (e.g., for the parse *clams*–*hell*, *clams* was seen as being related to *clamshell*), but as expected, the second, false constituent (e.g., *hell*) received a low rating.

### Measures of association (LSA and SNAUT)

Some researchers (e.g., Kuperman, 2013; Schmidtke, Gagné, Kuperman, & Spalding, 2018; Wang, Hsu, Tien, & Pomplun, 2014) have used measures of association as a proxy for semantic transparency. We used existing databases to retrieve measures of association for each constituent and the compound (e.g., *neck* and *necklace*, *lace* and *necklace*), as well as for the two constituents to each other (e.g., *neck* and *lace*). Although newer measures are becoming available (see, e.g., Gunther & Marelli, 2018), two of the most commonly used corpus-based measures of association within the compound literature thus far have been latent semantic analysis (LSA) and SNAUT, and thus we obtained two sets of measures: one based on LSA and one based on SNAUT. Both measures are based on co-occurrences. LSA is a method that analyzes large text corpora and derives the degree of association and semantic similarity from the use of the words in context (Landauer, 2002; Landauer & Dumais, 1997). LSA measures were obtained by using the pairwise comparison application on the LSA website (http://lsa.colorado.edu/) with General_Reading_up_to_1st_year_college (300 factors) as the topic space. Higher values indicate a higher degree of association. In addition, we used the SNAUT database (Mandera et al., 2017) to obtain the cosine distance as a measure of semantic association, via http://meshugga.ugent.be/snaut/. The cosine distance (which is 1 – cosine) is a measure of semantic dissimilarity: cosine distance equals 0 when the vectors are identical, 1 when the vectors are orthogonal, and 2 when the vectors are diametrically opposed. Thus, lower values indicate a higher degree of association. The descriptive statistics for these two sets of measures are provided in Table 3.

**Table 3:** Descriptive statistics for SNAUT cosine distance and latent semantic analysis (LSA) values

	*N*	Mean	*SD*	Min	Max
c1c2_snautCos	6,089	0.78	0.11	0.21	1.13
c1stim_snautCos	4,223	0.76	0.13	0.33	1.22
c2stim_snautCos	4,223	0.76	0.13	0.24	1.16
LSAc1c2	8,618	0.16	0.14	–0.13	1.00
LSAc1stim	4,473	0.17	0.18	–0.15	0.95
LSAc2stim	4,442	0.15	0.16	–0.17	0.94

### Sentiment and valence

A sentiment analysis for each constituent and compound was conducted in Mathematica using the Classify function. Sentiment measures were determined using a pretrained model that had been trained on social media text. The training set is appropriate for our participant population (who likely have daily exposure to such texts). The Classifier returns the probability that an item is Positive, Negative, Neutral, or Indeterminate, and uses these probabilities to place an item into one of these four categories. For example, *birthday*, *birth*, and *day* were all classified as Positive. *Weekend* and *week* were both Positive, whereas *end* was Negative. For each compound and for each constituent we also obtained the probability that the item is positive and the probability that the item is negative. We also calculated the ratio of the positive to negative probabilities. We also added a variable to indicate whether the compound and each constituent occurs in (Warriner et al., 2013). Their dataset contains valence, arousal, and dominance ratings for English words. The summary statistics for the valence (Warriner et al., 2013) and concreteness (Brysbaert et al., 2014) for the compound and each constituent are provided in Table 4.

**Table 4:** Descriptive statistics for sentiment (probability of positive sentiment, probability of negative sentiment, and ratio of positive to negative), valence (Warriner et al., 2013), and concreteness (Brysbaert et al., 2014)

	*N*	Mean	*SD*	Min	Max
Compound					
sentimentprobpos_stim	8,956	0.40	0.04	0.07	0.92
sentimentprobneg_stim	8,956	0.35	0.04	0.02	0.78
sentimentratioposneg_stim	8,956	1.17	0.69	0.09	55.36
valence_stim	2,213	5.19	1.19	1.59	8.30
concreteness_stim	4,647	3.98	0.78	1.27	5.00
First Constituent					
sentimentprobpos_c1	8,956	0.39	0.11	0.08	0.97
sentimentprobneg_c1	8,956	0.33	0.12	0.02	0.82
sentimentratioposneg_c1	8,956	1.43	0.90	0.22	49.06
valence_c1	7,952	5.58	1.15	1.43	8.37
concreteness_c1	8,383	4.17	0.82	1.43	5.00
Second Constituent					
sentimentprobpos_c2	8,956	0.38	0.10	0.12	0.84
sentimentprobneg_c2	8,956	0.34	0.10	0.04	0.76
sentimentratioposneg_c2	8,956	1.31	0.55	0.13	12.03
valence_c2	7,798	5.56	0.97	1.53	8.26
concreteness_c2	6,480	4.23	0.81	1.22	5.00

### Letter length

Length (in number of letters) of each compound and constituent is provided. The median lengths (with minimum and maximum values) for the full compound, first constituent, and second constituent of correctly parsed items are 9 (6–17), 4 (3–10), and 5 (3–10), respectively.

### Bigram frequency

Bigram frequency at the morpheme boundary has been shown to affect the processing of compound words (Gagne & Spalding, 2016). Therefore, we obtained two measures of bigram frequency for the letters at the morpheme boundary (e.g., the *l* and *p* in ballpark); one was retrieved from the table reported in Jones and Mewhort (2004), and the other was calculated on the basis of information provided in SUBTLEX-US. Bigram letter frequencies based on the SUBTLEX-US corpus were calculated using Mathematica, such that the bigram frequencies would accurately reflect exposure to the various letter combinations. This was done by taking the set of 74,286 words from SUBTLEX-US along with their frequency. An input set of words was created in which each word was replicated according to its frequency of occurrence. For example, the word *abandon* occurred 413 times in the SUBTLEX-US corpus and thus was repeated 413 times in the set. This set was used to determine all occurring letter bigrams along with their frequency. There were 600 unique bigrams contained in the SUBTLEX-US corpus with frequency counts ranging from 1 to 4,765,330. The two measures of bigram frequency (after log transforming the SUBTLEX-US measure) were highly correlated with each other (*r* = .95, *p* < .0001).

### Positional family size

Family size was defined as the number of compounds in the database that have the constituent of interest (e.g., the first constituent’s family size for *ball* includes *ballpark*, *ballgame*, *ballpen*, and *ballroom*, whereas the second constituent’s family size for *ball* includes *paintball*, *pinball*, *snowball*, and *hairball*). Our calculation of family size includes only correct parses. For example, *warpath* (*war* + *path*) is a family member of the first constituent *war*, but *wartweed* (which can be parsed as *war* + *tweed*) is not. The family sizes for the first constituent and second constituent were calculated. The median family sizes for the first constituent and second constituent were 8 (range 1–129) and 9 (range 1–228).

We also examined the relationship between positional family size and constituent frequency (as indexed by SUBTLEX-US’s logged frequency). As one would expect, positional family size is associated with that constituent’s family size for both the first and second constituents, in that higher frequencies are associated with larger family sizes (*r* = .498, *p* = .0025, and *r* = .487, *p* < .00001, respectively). Positional family size was also, but more weakly, associated with the other constituent’s frequency, in that larger family size was associated with lower frequency for the other constituent: *r* = – .065, *p* < .00001, for the first constituent’s family size and second constituent’s frequency, and *r* = – .168, *p* < .00001, for the second constituent’s family size and first constituent’s frequency.

### Profanity

The Classify function in Mathematica was used to classify each compound and each constituent in terms of whether it is considered profane. This includes swear words as well as words that are taboo or potentially offensive (e.g., *faggot* and *wetback*). The numbers of items identified as being profane, in terms of the compound, first constituent, and second constituent, were, in order, 13, 62, and 65.

### Plurality

The plurality of the compound was coded by the third author by initially using the ELP morphological parser and identifying strings in the MorphSp column ending with “>s>.” Any regular plural items that were missed by the ELP morphological parser, and all irregular plurals, were checked and coded manually.

### Availability in other corpora

We included indicator variables to indicate whether the items appear in other corpora, so that researchers can easily identify items that have information available in other sources. We included indicator variables to note whether the compound occurs in ELP and BLP, and whether each constituent occurs in ELP, Wordnet, and Mathematica’s Dictionary. Researchers can easily use the indicator variables when selecting stimuli, if they want to use other variables (such as frequency) available in these sources, by searching for items for which the relevant indicator variable (e.g., inELP or inBLP) is equal to 1. Information from the other corpora can easily be incorporated within researchers’ data management package of choice. For example, within Stata, the merge is straightforward and can be accomplished with a single command: *merge 1:1 stim using corpusname.dta*.

Another relevant corpus is Juhasz et al.’s (2015) set of 629 compounds. We included an indicator variable to denote which of our items appeared in their list. Of the 629 items in their list, 457 are part of our list of compounds. Some of the remaining items were compounds but were excluded due to not being nouns (e.g., *seasick* and *hitchhike*). Others were excluded because they were noncompounds and were formed via derivation or conversion (e.g., *censorship*, *fatherhood*, *fellowship*, *outlet*, *awesome*, *eyeless*, and *kinship*), were borrowings (e.g., *chamberlain*, *shamrock*, and *poppycock)*, or were monomorphemic*.*

We also included items from Kim et al.’s (2018) set, which includes items from Juhasz et al. (2015) and the ELP (Balota et al., 2007). The knowledge targeted by their ratings differs from either our or Juhasz et al.’s (2015) focus, in that participants were not directly asked about transparency or meaning retention. Instead, participants rated the relatedness of the meaning of the compound to either the first or the second constituent (e.g., *doorbell–door* or *doorbell*–*door*) on a scale from 1 (*not related*) to 7 (*highly related*). Each item was rated by 28–32 participants. Of Kim et al.’s items, 699 were not included in our original set of word–word items, due to not being nouns (e.g., *whoever*, *into*, *wouldbe*) or due to being proper names (e.g., *Bluebeard*, *Mayflower*). An additional 114 items did appear in our original set but were coded as noncompounds (e.g., *sainthood*, *priesthood*, *wardrobe*, *sponsorship*, *lordship*, *shamrock*, *bumpkin*).

To facilitate the selection of items that are in common usage or contain constituents that are in common usage, we included indicator variables denoting whether the compound and first and second constituents appear on a list of 40,127 common English words. This list is part of Mathematica’s built-in WordData package. Of the full set of 8,961 items (including incorrectly parsed items), 3,664 appear in the list of common words, 8,310 have first constituents appearing in the list of common words, and 6,599 have second constituents appearing in the list of common words. In terms of unique items (i.e., counting each parse only once, rather than counting all possible parses), 3,414 compounds appear on the list of common words.

In addition, we provide variables to indicate whether the frequency of a stimulus is available in the SUBTLEX-US corpus (Brysbaert & New, 2009) and in the Herdağdelen and Marelli (2017) social media frequency norms. The SUBTLEX-US corpus is a 51-million-token corpus based on subtitles from US film and media. The frequency norms derived from social media were based on the frequency per billion in the American corpus of 1.10 billion English posts on Facebook between November 2014 and January 2015. The Pearson correlation between these two measures of frequency (log-transformed) was .77 (*p* < .0001, *N* = 2,735). We also included as a variable SUBTLEX Zipf, which is a standardized measure of word frequency (van Heuven et al., 2014).

The summary statistics for log-transformed frequency from SUBTLEX-US, SUBTLEX Zipf, and the social media frequency norms, along with response times from the ELP and BLP, are provided in Table 5.

**Table 5:** Descriptive statistics for log word frequency from SUBTLEX, Zipf word frequency from SUBTLEX, log word frequency from Facebook data, lexical decision times from the English Lexicon Project (ELP) and British Lexicon Project (BLP), and naming times from ELP

	*N*	Mean	*SD*	Min	Max
Facebook frequency	3,202	1.91	0.97	–0.05	5.88
SUBTLEX frequency	5,308	1.06	0.62	0.30	4.36
SUBTLEX Zipf	5,308	2.35	0.62	1.59	5.65
ELP RT	3,148	806	126	534	1,588
BLP RT	2,609	685	82	431	1,274
ELP naming	3,149	716	91	546	1,158

## Analyses

To show the versatility of the database and determine whether the variables provide useful tools for understanding compound processing, we examined hypotheses ranging from orthographic to semantic issues. In particular, we tested hypotheses about how well the semantic transparency ratings correspond to existing ratings in the literature and whether our semantic transparency ratings, family size, morphemic boundary bigram frequency, and the various sentiment measures predict lexical decision and naming times.

### How are the meaning predictability and meaning retention ratings interrelated?

We had obtained three ratings per item. One, meaning predictability, focused on the overall transparency of the compound (e.g., the predictability of *eggplant* from *egg* and *plant*), and the other two, meaning retention for the first and second constituents (e.g., how much *egg* retains its meaning in *eggplant* and how much *plant* retains its meaning in *eggplant*), targeted the transparency of the individual constituents.

Given that all measures reflect some aspect of semantic transparency, it is useful to examine how these measures relate to each other. In our analysis, we focused on the 8,120 items that had correct parses (e.g., *eggs* + *hell* was excluded, but *egg* + *shell* was retained). The ratings of the meaning retention for the first and second constituents were correlated with each other, *r* = .31, *p* < .0001. Both ratings were associated with the compound-based meaning predictability measure. The meaning retention rating for the first constituent was correlated with the meaning predictability measure, *r* = .80, *p* < .0001, as was the meaning retention rating for the second constituent, *r* = .65, *p* < .0001. Steiger’s *z* test for correlations within a population (Steiger, 1980) indicates that the rating for the first constituent was more strongly correlated with the rating for the entire compound than was the rating for the second constituent, *z* = 27.71, *p* < .0001.

The finding that the overall semantic transparency rating is more strongly related for the first constituent than the second constituent is particularly interesting given that several theories of compounds emphasize the role of headedness. In English, the head is typically (with rare exceptions) the second constituent, and thus one might have expected to find the reverse of what we observed—namely, that the correlation between the second constituent’s and the compound’s transparency ratings would have been stronger than the correlation between the first constituent’s and the compounds transparency ratings. However, this finding fits well with previous research that has shown that the modifier (the first constituent in English) tends to play a larger role in the ease-of-relation selection during the processing of compounds and noun phrases (Gagné & Shoben, 1997; Spalding, Gagné, Mullaly, & Ji, 2010). We will return to this theoretical issue in the General Discussion.

### How associated are the present ratings of semantic transparency, Juhasz et al.’s (2015) ratings, Kim et al.’s (2018) ratings, and the corpus-based measures (SNAUT and LSA)?

Our next set of analyses examined the association between our semantic transparency rating and the rating presented in Juhasz et al. (2015), to better understand the relationship between the two sources of ratings, and also to examine whether ratings of the entire compound also reflect the transparency of the individual constituents. We also examined the semantic association ratings collected by Kim et al. (2018), as well as the corpus-based measures of association (i.e., LSA and SNAUT).

The Juhasz et al. (2015) measure (2015_trans) pertained to the entire compound: Participants were asked how transparent the meaning of the compound was on a scale ranging from 1–7 (with 7 being the highest level of transparency). The data were collected both individually and in a group setting via paper-and-pencil questionnaires. Each item was rated by between seven and 15 participants. In contrast, the items in present dataset were rated by a larger set of participants and were collected individually on a computer in a laboratory setting. Thus, it is useful to see how well these ratings correspond to each other. In addition to examining whether their rating corresponds to the rating of meaning predictability (e.g., how much the meaning of blueberry can be predicted from blue and berry), we can also examine how closely this compound-based judgment corresponds to the constituent-specific transparency judgments (e.g., how much *blue* or *berry* retains its meaning in *blueberry*).

To examine this question, we focused on the 456 words that were in both databases, and only on items that were classified as compounds in our database (e.g., items such as *friendship* and *outlet*, from Juhasz et al., 2015, were excluded). Information for all variables in this analysis was available for 429 words. As is shown in Table 6, the various judgments are significantly correlated with each other. The two measures that reflect compound-based transparency (i.e., the transparency rating from Juhasz et al., 2015, and the meaning predictability measure) exhibit the highest correlation. Both of these measures correlate with the constituent-specific ratings. Thus, Juhsasz et al.’s (2015) transparency measure and our compound-based measure reflect three sources of transparency: the overall transparency of the compound, as well as the transparency of the constituents.

**Table 6:** Pearson correlations among Juhasz et al.’s (2015) semantic transparency ratings (2015_trans); Kim et al.’s (2018) ratings for the first and second constituents (2018_ratingC1 and 2018_rating_C2); semantic transparency ratings for the first constituent (ratingC1), second constituent (ratingC2), and compound (ratingcmp); and SNAUT cosine distance and latent semantic analysis (LSA) values

	ratingcmp	ratingC1	ratingC2	2015 Trans	2018 transC1	2018 transC2	c1c2 snautCos	c1stim snautCos	c2stim snautCos	LSAc1c2	LSAc1stim
ratingC1	.75^***^	1.00									
ratingC2	.66^***^	.26^***^	1.00								
2015_trans	.86^***^	.73^***^	.68^***^	1.00							
2018_ratingC1	.62^***^	.77^***^	.23^***^	.67^***^	1.00						
2018_ratingC2	.47^***^	.17^***^	.72^***^	.53^***^	.26^***^	1.00					
c1c2_snautCos	– .26^***^	– .17^***^	– .18^***^	– .23^***^	– .16^***^	– .23^***^	1.00				
c1stim_snautCos	– .43^***^	– .51^***^	– .19^***^	– .49^***^	– .60^***^	– .21^***^	.24^***^	1.00			
c2stim_snautCos	– .26^***^	– .07	– .43^***^	– .33^***^	– .09	– .55^***^	.30^***^	.31^***^	1.00		
LSAc1c2	.19^***^	.12^*^	.10^*^	.12^*^	.04	.12^*^	– .71^***^	– .12^**^	– .18^***^	1.00	
LSAc1stim	.36^***^	.38^***^	.20^***^	.38^***^	.47^***^	.20^***^	– .15^**^	– .58^***^	– .21^***^	.11^*^	1.00
LSAc2stim	.21^***^	.04	.34^***^	.23^***^	.04	.43^***^	– .20^***^	– .13^**^	– .50^***^	.22^***^	.29^***^
*N*	429										

^*^p < .05, ^**^p < .01, ^***^p < .001.

Kim et al. (2018) did not specifically ask participants about transparency for their ratings, but rather about the degree of relatedness, which is a more general construct, in that it can be applied to any pair of words (e.g., *cat* and *milk*), not just to compounds and their constituents. Nonetheless, as can be seen in Table 6, their measures (2018_ratingC1 and 2018_ratingC2) do correlate with the other two sources of human ratings and, in particular, are most strongly correlated with the corresponding measure (e.g., the first-constituent ratings in Kim et al.’s study correlate more strongly with our first-constituent ratings than with our second-constituent ratings). This indicates that semantic relatedness is one aspect of semantic transparency.

In terms of the corpus-based measures, the LSA and SNAUT measures were also correlated with the human ratings to varying degrees. As one would expect, the correlation was strongest for the respective human ratings; for example, the measure of association between the first constituent and the compound correlated more highly with the meaning retention rating for the first constituent than with the meaning retention rating for the second constituent, whereas the reverse was true for the association between the second constituent and the compound. This pattern held for both our meaning retention measures and for Kim et al.’s (2018) ratings. Furthermore, the LSA and SNAUT measures were more strongly correlated with their respective ratings from Kim et al. than with the other two sets of human ratings (i.e., ours and Juhasz et al., 2015), which is to be expected, given that they specifically asked people to rate relatedness, which is the same construct that the LSA and SNAUT measures attempt to capture.

The Juhasz et al. (2015) semantic transparency rating was a single rating for the entire compound, and this rating was more strongly correlated with the association between the first constituent and the compound (as reflected by both the LSA and SNAUT measures) than with the association between the second constituent and the compound. The same pattern was observed for our meaning predictability rating (i.e., the degree to which the meaning of the compound is predictable from the first and second constituents). This might appear to be counterintuitive, but note that the meaning of the modifier more strongly relates to the difference between the meaning of the compound and the meaning of the head. That is, it is the modifier that changes the meaning of the compound away from the head (e.g., it is *snow* in *snowball* that makes it different from other balls). This is consistent with previous research showing that the ease of processing compounds is more strongly influenced by the modifier than by the head noun (Gagné & Shoben, 1997; Gagné & Spalding, 2014).

### Do human judgments of semantic transparency and relatedness influence ease of processing?

In this section, we examine whether the semantic transparency ratings are useful for predicting ease of processing. We will examined this issue by predicting lexical decision response times from the ELP (Balota et al., 2007), naming times from the ELP, and lexical decision times from the BLP (Keuleers et al., 2012). The response times in all models were log-transformed. Only the data corresponding to the correct parse were included (e.g., *work* + *space*, not *works* + *pace*).

Each of the three dependent variables was fit in separate sets of analyses. In terms of the control variables, our aim was to keep the models as simple as possible, to focus more directly on our variables of interest (i.e., the various semantic transparency measures) and to avoid issues of multicollinearity among the control variables. We began by fitting a baseline model that included the word length (i.e., number of letters) of the compound and the log word frequency of the compound in SUBTLEX-US as covariates. Then we fit a series of models in which we added in various rating measures to determine whether they successfully predicted response time. Model 2 included the control variables plus the Juhasz et al. (2015) transparency rating. Model 3 included the control variables plus Kim et al.’s (2018) relatedness ratings. Model 4 included the control variables plus the compound-based transparency rating (i.e., meaning predictability). Model 5 included all of the variables in Model 4 plus the two constituent-specific rating models. All four models were based only on the compounds that were in both Juhasz et al. (2015) and the present dataset. To determine whether the results using our three measures of transparency ratings generalized to a larger set of items, we fit two additional models for each dependent variable (i.e., ELP lexical decision, ELP naming time, and BLP lexical decision). Model 6 used the same variables as Model 4, but with all items in our database that had response times available from these three sources. Model 7 included the base model plus the constituent-based transparency measures.

In addition, we conducted a similar set of analyses, but also included length and frequency information about the constituents. In this second set of analyses, the length of the compound was not included, because the information provided by this variable is redundant with the length of the two constituents.

#### Results and discussion

The standardized coefficients, fit statistics, and sample sizes for predicting the ELP lexical decision times, BLP lexical decision times, and ELP naming times are shown in Tables 7, 8, and 9, respectively, for the models with the compound-based control variables, and in Tables 10, 11, and 12, for the models that also included constituent-based control variables. For the BLP data, frequency was represented either by SUBTLEX-US, to be consistent with the measure used in the analyses for the ELP data (top part of Tables 8 and 11), or by the BNC frequency (bottom part of Tables 8 and 11), to be consistent with the measure of frequency used in the original BLP article (Keuleers et al., 2012).

**Table 7:** Standardized regression coefficients with standard errors (in parentheses) from models using the semantic transparency measures and compound-based covariates to predict English Lexicon Project lexical decision times

	Model 1	Model 2	Model 3	Model 4	Model 5	Model 6	Model 7
SUBTLEX frequency	– 0.53^***^	– 0.52^***^	– 0.51^***^	– 0.51^***^	– 0.49^***^	– 0.45^***^	– 0.49^***^
	(0.00381)	(0.00382)	(0.00381)	(0.00380)	(0.00383)	(0.00161)	(0.00158)
stimlen	0.15^***^	0.15^***^	0.15^***^	0.15^***^	0.15^***^	0.23^***^	0.22^***^
	(0.00179)	(0.00179)	(0.00178)	(0.00177)	(0.00176)	(0.000654)	(0.000666)
2015_trans		– 0.03					
		(0.00190)					
2018_ratingC1			– 0.04				
			(0.00275)				
2018_ratingC2			– 0.08^*^				
			(0.00272)				
ratingcmp				– 0.12^**^	– 0.37^***^	– 0.38^***^	
				(0.000135)	(0.000305)	(0.000132)	
ratingC1					0.22^**^	0.16^***^	– 0.08^***^
					(0.000192)	(0.0000879)	(0.0000511)
ratingC2					0.12^*^	0.15^***^	– 0.04^*^
					(0.000189)	(0.0000837)	(0.0000571)
*N*	456	456	456	456	456	2513	2513
adj. R-sq	0.308	0.308	0.316	0.322	0.334	0.349	0.324
AIC	– 1,494.9	– 1,493.6	– 1,498.0	– 1,503.0	– 1,509.4	– 8,065.1	– 7,969.7

Standardized beta coefficients; standard errors are in parentheses. ^*^p < .05, ^**^p < .01, ^***^p < .001.

**Table 8:** Standardized regression coefficients with standard errors (in parentheses) from models using the semantic transparency measures and compound-based covariates to predict British Lexicon Project lexical decision times

	Model 1	Model 2	Model 3	Model 4	Model 5	Model 6	Model 7
Analyses using SUBTLEX to estimate frequency							
SUBTLEX frequency	– 0.52^***^	– 0.52^***^	– 0.51^***^	– 0.50^***^	– 0.50^***^	– 0.43^***^	– 0.47^***^
	(0.00375)	(0.00370)	(0.00369)	(0.00367)	(0.00371)	(0.00146)	(0.00144)
stimlen	– 0.06	– 0.06	– 0.05	– 0.05	– 0.06	0.02	– 0.00
	(0.00220)	(0.00217)	(0.00216)	(0.00215)	(0.00214)	(0.000766)	(0.000775)
2015_trans		– 0.14^**^					
		(0.00184)					
2018_ratingC1			– 0.09				
			(0.00266)				
2018_ratingC2			– 0.14^**^				
			(0.00265)				
ratingcmp				– 0.19^***^	– 0.33^**^	– 0.40^***^	
				(0.000131)	(0.000310)	(0.000122)	
ratingC1					0.17^*^	0.19^***^	– 0.06^**^
					(0.000196)	(0.0000812)	(0.0000477)
ratingC2					0.01	0.14^***^	– 0.06^**^
					(0.000189)	(0.0000789)	(0.0000538)
*N*	319	319	319	319	319	1999	1999
adj. R-sq	0.262	0.281	0.291	0.298	0.305	0.262	0.233
AIC	– 1,161.7	– 1,168.9	– 1,172.4	– 1,176.4	– 1,177.8	– 7,163.9	– 7,089.9
Analyses using SUBTLEX to estimate frequency							
BNC frequency	– 0.56^***^	– 0.57^***^	– 0.56^***^	– 0.56^***^	– 0.56^***^	– 0.44^***^	– 0.47^***^
	(0.00307)	(0.00300)	(0.00300)	(0.00295)	(0.00295)	(0.00127)	(0.00128)
stimlen	– 0.01	– 0.01	– 0.00	– 0.01	– 0.02	0.07^***^	0.05^**^
	(0.00213)	(0.00207)	(0.00208)	(0.00204)	(0.00203)	(0.000726)	(0.000744)
2015_trans		– 0.19^***^					
		(0.00177)					
2018_transC1			– 0.09				
			(0.00255)				
2018_transC2			– 0.16^**^				
			(0.00256)				
ratingcmp				– 0.24^***^	– 0.42^***^	– 0.46^***^	
				(0.000125)	(0.000291)	(0.000115)	
ratingC1					0.21^*^	0.23^***^	– 0.05^**^
					(0.000185)	(0.0000785)	(0.0000476)
ratingC2					0.04	0.14^***^	– 0.08^***^
					(0.000178)	(0.0000769)	(0.0000535)
* N*	319	319	319	319	319	2392	2392
adj. R-sq	0.307	0.342	0.343	0.362	0.374	0.285	0.247
AIC	– 1,181.7	– 1,197.1	– 1,196.9	– 1,207.1	– 1,210.9	– 8,366.6	– 8,245.4

Standardized beta coefficients; standard errors are in parentheses. ^*^p < .05, ^**^p < .01, ^***^p < .001

**Table 9:** Standardized regression coefficients with standard errors (in parentheses) from models using the semantic transparency measures and compound-based covariates to predict English Lexicon Project naming times

	Model 1	Model 2	Model 3	Model4	Model 5	Model 6	Model 7
SUBTLEX frequency	– 0.47^***^	– 0.47^***^	– 0.47^***^	– 0.46^***^	– 0.44^***^	– 0.38^***^	– 0.42^***^
	(0.00325)	(0.00326)	(0.00326)	(0.00324)	(0.00327)	(0.00135)	(0.00131)
stimlen	0.25^***^	0.25^***^	0.25^***^	0.26^***^	0.25^***^	0.29^***^	0.28^***^
	(0.00152)	(0.00153)	(0.00152)	(0.00151)	(0.00150)	(0.000548)	(0.000554)
2015_trans		– 0.02					
		(0.00162)					
2018_ratingC1			– 0.00				
			(0.00236)				
2018_ratingC2			– 0.08				
			(0.00232)				
ratingcmp				– 0.12^**^	– 0.34^***^	– 0.33^***^	
				(0.000115)	(0.000261)	(0.000111)	
ratingC1					0.20^**^	0.11^***^	– 0.10^***^
					(0.000164)	(0.0000737)	(0.0000425)
ratingC2					0.11	0.14^***^	– 0.02
					(0.000162)	(0.0000701)	(0.0000476)
* N*	456	456	456	456	456	2514	2514
adj. R-sq	0.305	0.303	0.307	0.317	0.327	0.304	0.286
AIC	– 1,640.6	– 1,638.8	– 1,640.5	– 1,648.0	– 1,652.4	– 8,957.4	– 8,893.1

Standardized beta coefficients; Standard errors are in parentheses. ^*^p < .05, ^**^p < .01, ^***^p < .001.

**Table 10:** Standardized regression coefficients with standard errors (in parentheses) from models using the semantic transparency measures, compound-based covariates, and constituent-based covariates to predict English Lexicon Project lexical decision times

	Model 1	Model 2	Model 3	Model 4	Model 5	Model 6	Model 7
Frequency	– 0.48^***^	– 0.48^***^	– 0.46^***^	– 0.47^***^	– 0.45^***^	– 0.42^***^	– 0.46^***^
	(0.00393)	(0.00394)	(0.00394)	(0.00392)	(0.00393)	(0.00163)	(0.00160)
c1_SLlg10wf	– 0.06	– 0.06	– 0.07	– 0.05	– 0.06	– 0.11^***^	– 0.11^***^
	(0.00274)	(0.00275)	(0.00273)	(0.00274)	(0.00272)	(0.00128)	(0.00130)
c2_SLlg10wf	– 0.15^***^	– 0.15^***^	– 0.17^***^	– 0.15^***^	– 0.14^***^	– 0.09^***^	– 0.09^***^
	(0.00285)	(0.00285)	(0.00287)	(0.00283)	(0.00281)	(0.00114)	(0.00116)
c1len	0.16^***^	0.16^***^	0.15^***^	0.16^***^	0.16^***^	0.20^***^	0.20^***^
	(0.00221)	(0.00221)	(0.00219)	(0.00219)	(0.00218)	(0.000928)	(0.000943)
c2len	0.00	0.00	0.00	0.01	0.01	0.09^***^	0.07^***^
	(0.00280)	(0.00280)	(0.00278)	(0.00279)	(0.00276)	(0.000992)	(0.00100)
2015_trans		– 0.03					
		(0.00187)					
2018_ratingC1			– 0.04				
			(0.00270)				
2018_ratingC2			– 0.11^**^				
			(0.00271)				
ratingcmp				– 0.11^**^	– 0.33^***^	– 0.35^***^	
				(0.000134)	(0.000302)	(0.000131)	
ratingC1					0.21^**^	0.16^***^	– 0.06^***^
					(0.000189)	(0.0000875)	(0.0000512)
ratingC2					0.10	0.14^***^	– 0.03
					(0.000187)	(0.0000831)	(0.0000568)
* N*	456	456	456	456	456	2501	2501
adj. R-sq	0.335	0.334	0.347	0.345	0.356	0.369	0.348
AIC	– 1,509.7	– 1,508.5	– 1,516.4	– 1,515.7	– 1,521.3	– 8,107.1	– 8,024.5

Standardized beta coefficients; Standard errors are in parentheses. ^*^p < .05, ^**^p < .01, ^***^p < .001.

**Table 11:** Standardized regression coefficients with standard errors (in parentheses) from models using the semantic transparency measures, compound-based covariates, and constituent-based covariates to predict British Lexicon Project lexical decision times

	Model 1	Model 2	Model 3	Model4	Model 5	Model 6	Model 7
Analyses using SUBTLEX to estimate frequency							
SUBTLEX frequency	– 0.48^***^	– 0.48^***^	– 0.46^***^	– 0.47^***^	– 0.47^***^	– 0.42^***^	– 0.46^***^
	(0.00393)	(0.00388)	(0.00385)	(0.00384)	(0.00387)	(0.00150)	(0.00149)
c1_SLlg10wf	– 0.02	– 0.02	– 0.04	– 0.00	– 0.01	– 0.02	– 0.03
	(0.00268)	(0.00265)	(0.00263)	(0.00263)	(0.00262)	(0.00118)	(0.00121)
c2_SLlg10wf	– 0.12^*^	– 0.13^*^	– 0.16^**^	– 0.13^*^	– 0.12^*^	– 0.06^**^	– 0.05^*^
	(0.00299)	(0.00295)	(0.00298)	(0.00292)	(0.00292)	(0.00114)	(0.00116)
c1len	– 0.01	– 0.01	0.00	– 0.01	– 0.01	0.02	0.01
	(0.00306)	(0.00302)	(0.00298)	(0.00299)	(0.00299)	(0.00113)	(0.00115)
c2len	– 0.10^*^	– 0.10^*^	– 0.10^*^	– 0.09	– 0.10	– 0.02	– 0.04
	(0.00312)	(0.00308)	(0.00304)	(0.00305)	(0.00304)	(0.00113)	(0.00114)
2015_trans		– 0.15^**^					
		(0.00183)					
2018_ratingC1			– 0.09				
			(0.00264)				
2018_ratingC2			– 0.17^***^				
			(0.00268)				
ratingcmp				– 0.19^***^	– 0.30^**^	– 0.42^***^	
				(0.000131)	(0.000311)	(0.000122)	
ratingC1					0.15	0.21^***^	– 0.05^*^
					(0.000196)	(0.0000810)	(0.0000480)
ratingC2					– 0.00	0.15^***^	– 0.06^**^
					(0.000189)	(0.0000790)	(0.0000540)
*N*	319	319	319	319	319	1994	1994
adj. R-sq	0.271	0.292	0.310	0.306	0.312	0.270	0.240
AIC	– 1,162.7	– 1,170.8	– 1,178.1	– 1,177.2	– 1,177.9	– 7,181.4	– 7,102.1
Analyses using SUBTLEX to estimate frequency							
BNC frequency	– 0.51^***^	– 0.53^***^	– 0.50^***^	– 0.53^***^	– 0.53^***^	– 0.43^***^	– 0.46^***^
	(0.00329)	(0.00322)	(0.00319)	(0.00317)	(0.00317)	(0.00134)	(0.00136)
c1_BNC frequency	– 0.06	– 0.04	– 0.06	– 0.03	– 0.03	– 0.04	– 0.04^*^
	(0.00285)	(0.00279)	(0.00277)	(0.00277)	(0.00275)	(0.00127)	(0.00130)
c2_BNC frequency	– 0.10^*^	– 0.10^*^	– 0.13^**^	– 0.09	– 0.08	– 0.04^*^	– 0.03
	(0.00314)	(0.00307)	(0.00310)	(0.00303)	(0.00303)	(0.00124)	(0.00127)
c1len	0.02	0.02	0.03	0.02	0.01	0.05^*^	0.05^**^
	(0.00298)	(0.00291)	(0.00289)	(0.00287)	(0.00286)	(0.00109)	(0.00111)
c2len	– 0.06	– 0.05	– 0.05	– 0.04	– 0.05	0.04^*^	0.02
	(0.00295)	(0.00288)	(0.00286)	(0.00285)	(0.00283)	(0.00102)	(0.00105)
2015_trans		– 0.19^***^					
		(0.00177)					
2018_ratingC1			– 0.09				
			(0.00254)				
2018_ratingC2			– 0.18^***^				
			(0.00258)				
ratingcmp				– 0.23^***^	– 0.39^***^	– 0.46^***^	
				(0.000126)	(0.000293)	(0.000117)	
ratingC1					0.20^*^	0.24^***^	– 0.04^*^
					(0.000186)	(0.0000800)	(0.0000488)
ratingC2					0.03	0.14^***^	– 0.08^***^
					(0.000178)	(0.0000789)	(0.0000549)
*N*	319	319	319	319	319	2360	2360
adj. R-sq	0.316	0.348	0.358	0.365	0.375	0.286	0.249
AIC	– 1,182.9	– 1,197.4	– 1,201.4	– 1,205.4	– 1,208.6	– 8,241.8	– 8,122.9

Standardized beta coefficients; standard errors are in parentheses. ^*^p < .05, ^**^p < .01, ^***^p < .001

**Table 12:** Standardized regression coefficients with standard errors (in parentheses) from models using semantic transparency measures compound-based covariates and constituent-based covariates to predict English Lexicon Project naming times

	Model 1	Model 2	Model 3	Model 4	Model 5	Model 6	Model 7
SUBTLEX frequency	– 0.40^***^	– 0.40^***^	– 0.38^***^	– 0.39^***^	– 0.38^***^	– 0.33^***^	– 0.36^***^
	(0.00326)	(0.00327)	(0.00328)	(0.00325)	(0.00327)	(0.00132)	(0.00129)
c1_SLlg10wf	– 0.19^***^	– 0.19^***^	– 0.19^***^	– 0.18^***^	– 0.18^***^	– 0.22^***^	– 0.22^***^
	(0.00228)	(0.00228)	(0.00227)	(0.00227)	(0.00226)	(0.00104)	(0.00105)
c2_SLlg10wf	– 0.14^***^	– 0.14^***^	– 0.16^***^	– 0.14^***^	– 0.13^***^	– 0.11^***^	– 0.12^***^
	(0.00236)	(0.00236)	(0.00239)	(0.00235)	(0.00234)	(0.000924)	(0.000933)
c1len	0.24^***^	0.24^***^	0.23^***^	0.24^***^	0.23^***^	0.23^***^	0.23^***^
	(0.00183)	(0.00183)	(0.00182)	(0.00182)	(0.00181)	(0.000753)	(0.000761)
c2len	0.05	0.05	0.05	0.06	0.06	0.12^***^	0.11^***^
	(0.00232)	(0.00233)	(0.00231)	(0.00231)	(0.00230)	(0.000805)	(0.000810)
2015_trans		– 0.02					
		(0.00155)					
2018_ratingC1			– 0.00				
			(0.00225)				
2018_ratingC2			– 0.10^**^				
			(0.00225)				
ratingcmp				– 0.09^*^	– 0.29^***^	– 0.28^***^	
				(0.000111)	(0.000251)	(0.000107)	
ratingC1					0.19^**^	0.12^***^	– 0.06^***^
					(0.000158)	(0.0000710)	(0.0000413)
ratingC2					0.09	0.12^***^	– 0.02
					(0.000156)	(0.0000675)	(0.0000459)
* N*	456	456	456	456	456	2502	2502
adj. R-sq	0.367	0.366	0.375	0.374	0.383	0.364	0.351
AIC	– 1,680.8	– 1,678.9	– 1,684.5	– 1,684.7	– 1,689.1	– 9,153.2	– 9,103.1

Standardized beta coefficients; standard errors are in parentheses. ^*^p < .05, ^**^p < .01, ^***^p < .001.

The transparency ratings from Juhasz et al. (2015) did not successfully predict ELP lexical decision times (Model 2, Table 7) but did predict BLP lexical decision times (Model 2, Table 8). The Kim et al. (2018) rating for the second constituent was a successful predictor of both ELP (Model 3, Table 7) and BLP (Model 3, Table 8) lexical decision times, but the rating for the first constituent was not predictive. The meaning predictability rating and the meaning retention rating for the first constituent from the present study successfully predicted responses in all models (i.e., for ELP and BLP times, and for the reduced and full set of compounds; Models 4–7), and the meaning retention rating for the second constituent was also a successful predictor in all models except Model 5 (Table 8) for the BLP lexical decision times.

Neither the Juhasz et al. (2015) ratings (Model 2, Table 9) nor the Kim et al. (2018) ratings (Model 3, Table 9) predicted naming times. In contrast, meaning predictability and meaning retention for the first constituent were successful predictors in all models. Meaning retention for the second constituent was a successful predictor only for the full item set when meaning predictability was also included in the model (Model 6, Table 9).

The models that included the frequency and letter length of the constituents show patterns identical to those of the models reported in the previous paragraphs, except that the meaning retention ratings for the second constituent for the model predicting BLP lexical decision for the reduced set of items (Model 5, Table 11) were not predictive, nor were the meaning retention ratings for the second constituent successful in predicting ELP lexical decision times for the reduced set of items (Model 5, Table 10). Another difference to note was that the meaning retention of C1 emerged as a valid predictor of the reduced set of compounds when compound frequency was estimated using BNC (Model 5, Table 11). Kim et al.’s (2018) rating for the second constituent emerged as a valid predictor of naming times when covariates for the constituents were included (Model 3, Table 11).

In sum, these analyses indicate that the present set of semantic transparency ratings more consistently predicted ease of processing than did the Juhasz et al. (2015) rating, which was a successful predictor of one (BLP lexical decision time) out of the three processing measures. Also, the present set of ratings detected the influence of the first constituent, whereas the relatedness ratings in Kim et al. (2018) only showed the influence of the second constituent. Recall that our measure asked participants how much of the meaning of a constituent was retained, whereas Kim et al. asked people to rate how related the constituent and compound were. The present analyses suggest that response times are affected by how much of the meaning of *foot* is retained in *football*, but not by how much *foot* and *football* are related.

Overall, our models indicate that semantic transparency does influence ease of processing and that the nature of this influence depends on whether the transparency is based on the overall compound or the constituents, and on whether both constituent-based and compound-based measures are included in the model. Compounds with meanings that are more highly predictable from their constituents are processed more quickly than compounds that are less predictable from their constituent. That is, semantic transparency is beneficial. However, in terms of the constituent-specific measures, the coefficient was positive, indicating that increased ratings of retention were associated with slower times when the rating for the entire compound was included in the model (Models 5 and 6). However, when ratingcmp (the rating for the entire compound) was excluded (Model 7), higher ratings for the constituents were associated with faster response times. Thus, when both constituent-based and compound-based measures of transparency are included in the model, the constituent-based coefficients indicate slower responses when the ratings are higher. This pattern (i.e., the flip in direction of the coefficients for the constituents when the compound-based measure was removed from the analysis) held for both the ELP and BLP lexical decision times, as well as for the ELP naming times. As is indicated in Table 6, the meaning predictability rating is highly correlated with the meaning retention ratings for the first and second constituents (*r*s = .75 and .66), and thus they compete with each other as predictors. In the present models, meaning predictability (which takes into account the contribution of both constituents) represents the expected reciprocal relationship (in that higher ratings are associated with faster response times), with the other two ratings reflecting adjustments that are uniquely based on the constituents and not shared with meaning predictability.

## Do corpus-based measures of association influence ease of processing?

We evaluated whether the two types of corpus-based associations measures (LSA and SNAUT) predicted lexical decision (ELP and BLP) and naming (ELP) times using items for which both measures were available. As in the models with the human ratings, constituent- and compound-based control variables were also included. The output of these models is provided in Table 13. For the BLP data, frequency was represented by SUBTLEX-US and, in a separate model, by BNC frequency.

**Table 13:** Standardized regression coefficients with standard errors (in parentheses) from models using vector-based measures of semantic transparency to predict English Lexicon Project (ELP) lexical decision (LD) times, British Lexicon Project (BLP) lexical decision times, and ELP naming times

	ELP LD	ELP LD	BLP LD	BLP LD	BLP LD	BLP LD	ELP Naming	ELP Naming
SUBTLEX frequency	– 0.423^***^ (0.00190)	– 0.451^***^ (0.00191)	– 0.484^***^ (0.00197)	– 0.506^***^ (0.00197)			– 0.349^***^ (0.00154)	– 0.373^***^ (0.00153)
c1_SLlg10wf	– 0.143^***^ (0.00151)	– 0.138^***^ (0.00155)	– 0.034 (0.00164)	– 0.032 (0.00167)			– 0.248^***^ (0.00122)	– 0.244^***^ (0.00124)
c2_SLlg10wf	– 0.115^***^ (0.00138)	– 0.101^***^ (0.00141)	– 0.122^***^ (0.00154)	– 0.107^***^ (0.00160)			– 0.147^***^ (0.00111)	– 0.142^***^ (0.00113)
BNC_frequency					– 0.482^***^ (0.00186)	– 0.492^***^ (0.00180)		
c1_BNC frequency					– 0.028 (0.00178)	– 0.036 (0.00179)		
c2_BNC frequency					– 0.069^**^ (0.00170)	– 0.065^*^ (0.00173)		
c1len	0.180^***^ (0.00112)	0.179^***^ (0.00113)	0.026 (0.00152)	0.024(0.00152)	0.057^*^ (0.00153)	0.059^*^ (0.00152)	0.194^***^ (0.000904)	0.193^***^ (0.000903)
c2len	0.095^***^ (0.00116)	0.092^***^ (0.00116)	– 0.028 (0.00163)	– 0.029 (0.00163)	0.047 (0.00160)	0.045 (0.00159)	0.104^***^ (0.000932)	0.105^***^ (0.000931)
LSAc1c2	0.022 (0.00791)		0.005 (0.00866)		– 0.030 (0.00860)		0.033 (0.00638)	
LSAc1stim	– 0.072^***^ (0.00631)		– 0.064^*^ (0.00673)		– 0.055^*^(0.00679)		– 0.031 (0.00509)	
LSAc2stim	– 0.047^*^ (0.00729)		– 0.031 (0.00751)		– 0.043 (0.00749)		– 0.018 (0.00588)	
c1c2_snautCos		0.002 (0.0107)		0.014 (0.0119)		0.061^*^ (0.0113)		– 0.043 (0.00860)
c1stim_snautCos		0.025 (0.00956)		0.036 (0.0100)		0.091^***^ (0.00992)		– 0.007 (0.00765)
c2stim_snautCos		– 0.042^*^ (0.00915)		– 0.049 (0.00979)		0.024 (0.00969)		– 0.039 (0.00733)
* N*	1,767	1,767	1,121	1,121	1,191	1,191	1,768	1,768
R-sq	0.350	0.344	0.304	0.301	0.293	0.304	0.344	0.346
adj. R-sq	0.347	0.341	0.299	0.296	0.288	0.299	0.341	0.343
AIC	– 5,774.3	– 5,757.3	– 4,037.6	– 4,033.1	– 4,203.5	– 4,221.1	– 6,536.8	– 6,543.4

Standardized beta coefficients; standard errors are in parentheses. ^*^p < .05, ^**^p < .01, ^***^p < .001.

The LSA measures were successful at predicting lexical decision times, with the association between the first constituent and compound being a successful predictor of both the ELP and BLP lexical decision times, and the association between the second constituent and the compound being a successful predictor of ELP lexical decision times. In terms of the SNAUT measures, the association for the second constituent predicted ELP lexical decision times, but not BLP lexical decision times. None of the LSA or SNAUT measures were valid predictors of naming. In sum, the LSA measures were more consistent predictors across the three dependent variables than were the SNAUT measures.

It is worth noting that these corpus-based measures do not take into account the semantics of open compounds. Open compounds reflect the potentiality of the constituents’ ability to combine with other constituents during conceptual combination (see Gagné & Shoben, 1997, for a discussion of how people appear to have knowledge of how concepts are used to modify and be combined with other concepts). Indeed, Marelli, Dinu, Zamparelli, and Baroni (2015) found that distribution-based semantic transparency variables are more predictive of response times when derived from open-form compounds than when derived from closed-form compounds, which suggests that compound processing is compositional. Thus, their findings, as well as the present finding that LSA and SNAUT had limited predictive power, is consistent with previous evidence for the role of composition in compound processing (see Gagné & Spalding, 2014, for an overview).

### Does morpheme bigram frequency influence ease of processing?

To test whether ease of parsing influences ease of processing, we evaluated whether bigram frequency predicted lexical decision (ELP and BLP) and naming (ELP) times. We used both types of dependent variables to determine whether lexical decision is more likely to be influenced by bigram frequency than is naming. Naming is more remote from letter identification, and perhaps less susceptible to the orthographic properties of a word. In general, one would expect higher-frequency letter combinations to aid the identification and processing of words. However, if compound processing involves parsing, then words with higher bigram frequencies at the morpheme boundary (e.g., *th* in *anthill*) should be more difficult to process than words with lower bigram frequencies (e.g., *xg* in *foxglove*). In short, low bigram frequency could be an indicator of a likely parse.

For these analyses, we focused only the correctly parsed compounds. Word frequency and word length were entered as control variables. One set of models used bigram frequency measures obtained from Jones and Mewhort (2004). A second set of models used the bigram frequency calculated from SUBTLEX-US. We predicted the two lexical decision measures (ELP and BLP) and the ELP naming times in separate models. The standardized coefficients and model fits for these models are shown in Table 14. For the BLP data, frequency is represented by SUBTLEX-US and, in a separate model, by BNC frequency.

**Table 14:** Standardized regression coefficients with standard errors (in parentheses) from models using frequency, stimulus length (in letters), log bigram frequency (based either on SUBTLEX or from Jones & Mewhort, 2004), to predict English Lexicon Project (ELP) lexical decision (LD) times, British Lexicon Project (BLP) lexical decision times, and ELP naming times

	ELP LD	ELP LD	BLP LD	BLP LD	BLP LD	BLP LD	ELP Naming	ELP Naming
SUBTLEX frequency	– 0.491^***^	– 0.491^***^	– 0.479^***^	– 0.478^***^			– 0.415^***^	– 0.414^***^
	(0.00156)	(0.00156)	(0.00144)	(0.00144)			(0.00131)	(0.00131)
BNC frequency					– 0.486^***^	– 0.486^***^		
					(0.00129)	(0.00129)		
stimlen	0.220^***^	0.217^***^	– 0.015	– 0.015	0.042^*^	0.042^*^	0.258^***^	0.256^***^
	(0.000645)	(0.000646)	(0.000774)	(0.000774)	(0.000743)	(0.000743)	(0.000542)	(0.000543)
bgSUBTLEX	0.078^***^		0.038		0.041^*^		0.087^***^	
	(0.000975)		(0.000902)		(0.000852)		(0.000819)	
bgJonesMewhort		0.088^***^		0.035		0.041^*^		0.087^***^
		(0.000460)		(0.000422)		(0.000399)		(0.000387)
* N*	2,593	2,593	2,002	2,002	2,396	2,396	2,594	2,594
R-sq	0.322	0.324	0.226	0.226	0.239	0.239	0.273	0.273
adj. R-sq	0.321	0.323	0.225	0.225	0.238	0.238	0.272	0.272
AIC	– 8,199.6	– 8,205.8	– 7,079.1	– 7,078.6	– 8,231.6	– 8,231.4	– 9,106.2	– 9,106.3

Standardized beta coefficients; standard errors are in parentheses. ^*^p < .05, ^**^p < .01, ^***^p < .001.

The pattern of results is consistent across both methods of obtaining bigram frequency. Both measures were valid predictors of ELP lexical decision and naming times, but not of BLP lexical decision times when compound frequency was estimated by SUBTLEX-US (the same measure used to estimate the ELP data), but the ratings were valid predictors of BLP lexical decision time when word frequency was estimated using BNC frequency.

To examine whether the impact of bigram frequency is influenced by whether the recovered morphemes are productive (i.e., whether or not they function as morphemes in the word), we refit the models but allowed bigram frequency to interact with a variable indicating whether the item was correctly parsed (e.g., *wartweed* –> *wart*–*weed*, where the bigram of interest is *tw*) or not (e.g., *war*–*tweed*, where the bigram of interest is *rt*). We present only the bigram measure based on SUBTLEX-US in Table 15 because both measures of bigram frequencies showed consistent patterns. As can be seen there, bigram frequency at the boundary interacted with whether the item was correctly parsed for the two measures of lexical decision, but not for the naming latencies, which suggests that naming is less sensitive than lexical decision to the orthographic characteristics of the stimulus.

**Table 15:** Standardized regression coefficients with standard errors (in parentheses) from models using frequency, stimulus length (in letters), log bigram frequency calculated from SUBTLEX, and whether the item was correctly parsed or not to predict English Lexicon Project (ELP) lexical decision (LD) times, British Lexicon Project (BLP) lexical decision times, and ELP naming times

	ELP LD	BLP LD	BLP LD	ELP Naming
SUBTLEX frequency	– 0.487^***^	– 0.472^***^		– 0.409^***^
	(0.00152)	(0.00140)		(0.00128)
BNC frequency			– 0.482^***^	
			(0.00125)	
stimlen	0.217^***^	– 0.010	0.048^**^	0.264^***^
	(0.000627)	(0.000753)	(0.000723)	(0.000527)
log_bgSUBTLEX	– 0.335^*^	– 0.476^**^	– 0.409^**^	– 0.085
	(0.00801)	(0.00693)	(0.00660)	(0.00673)
Correct parse	– 0.561^**^	– 0.788^***^	– 0.652^**^	– 0.208
	(0.0459)	(0.0401)	(0.0380)	(0.0386)
Bigram frequency x Correct parse	0.244^**^	.023^**^	0.021	0.008
	(0.00807)	(0.00698)	(0.00665)	(0.00678)
* N*	2,773	2,183	2,604	2,774
adj. R-sq	0.318	0.226	0.240	0.270
AIC	– 8,762.3	– 7,704.8	– 8,929.3	– 9,730.1

Standardized beta coefficients; standard errors are in parentheses. ^*^p < .05, ^**^p < .01, ^***^p < .001.

Due to the interaction in the two models predicting lexical decision, we examined the influence of bigram frequency separately for the correctly and incorrectly parsed compounds using a simple-effects analysis. In the model predicting ELP lexical decision times, high bigram frequency was associated with longer response times for the correctly parsed items, *b* = 0.00465, *se* = .001, *t* = 4.77, *p* < .0001. In contrast, for incorrectly parsed items, high bigram frequency was associated with faster responses times, *b* = – 0.0198, *se* = .008, *t* = *–* 2.46, *p* = .014. The model predicting BLP lexical decision times also revealed that higher bigram frequencies were associated with slower response times for correctly parsed compounds, *b* = .0017, *se* = .0009, *t* = 1.89, *p* = .06, but were associated with faster response times for incorrectly parsed compounds, *b* = – .021, *se* = .007, *t* = *–* 3.05, *p* = .002.

In sum, these analyses reveal that lexical decision is sensitive to the frequency of bigrams at the edges of embedded words (e.g., the frequency of the *ks* in *work-space* and *sp* in *works-pace*), but the nature of this influence depends on whether the embedded words are morphemic constituents of the compound (e.g., *boot-hose*; *cow-slip*) or not (e.g., *boo-those*; *cows-lip*). When the bigram is at a true morpheme boundary, higher frequencies make it more difficult to parse and identify the two constituents, which increases processing time. However, when the bigram is not at a morpheme boundary, high frequencies aid in letter identification, which aids access to the compound.

### Does family size influence ease of processing?

We evaluated whether our family size measures predicted lexical decision (ELP and BLP) and naming times (ELP). As in the previous section, word frequency and word length were entered as control variables. Only items with the correct parse and with at least one family member were included in the analysis. A summary of the models is shown in Table 16. The number of compounds using the same first constituent successfully predicts ELP and BLP lexical decision times as well as naming times. The greater the number of family members with the same first constituent, the easier it was to process the compound. In contrast, the number of compounds using the same second constituent was not a successful predictor of naming or of lexical decision times. This pattern is similar to that from our analysis of the correlation between the constituent and whole-word transparency measures, in which the first constituent transparency rating was more highly correlated with the whole-word transparency measure than was the second constituent transparency measure. Taken together, these analyses suggest a special role for the first constituent in compound-word processing. We return to this issue in the General Discussion.

**Table 16:** Standardized regression coefficients with standard errors (in parentheses) from models using frequency, stimulus length (in letters), log bigram frequency, family size of the first constituent, and family size of the second constituent to predict English Lexicon Project (ELP) lexical decision (LD) times, British Lexicon Project (BLP) lexical decision times, and ELP naming times

	ELP LD	BLP LD	BLP LD	ELP Naming
SUBTLEX frequency	– 0.481^***^	– 0.469^***^		– 0.397^***^
	(0.00155)	(0.00143)		(0.00127)
BNC frequency			– 0.472^***^	
			(0.00129)	
stimlen	0.227^***^	– 0.031	0.034	0.263^***^
	(0.000645)	(0.000787)	(0.000758)	(0.000525)
nc1_cmp	– 0.117^***^	– 0.122^***^	– 0.098^***^	– 0.242^***^
	(0.0000394)	(0.0000506)	(0.0000490)	(0.0000321)
nc2_cmp	0.005	– 0.038	– 0.008	– 0.024
	(0.0000206)	(0.0000198)	(0.0000191)	(0.0000168)
* N*	2,593	2,002	2,396	2,594
adj. R-sq	0.329	0.238	0.246	0.322
AIC	– 8,227.7	– 7,112.9	– 8,253.5	– 9,288.9

Standardized beta coefficients; standard errors are in parentheses. ^*^p < .05, ^**^p < .01, ^***^p < .001.

### Are lexical decision and naming sensitive to the sentiment and valence of the compound and its constituents?

We examined whether the sentiment of the compound and its constituents influences ease of processing, as indexed by lexical decision times in the ELP (Balota et al., 2007) and BLP (Keuleers et al., 2012) and by naming latencies in the ELP. We fit one model for each of these three dependent variables. In all models, word length (i.e., number of letters) and log word frequency in SUBTLEX-US were entered as control variables. As predictor variables, we entered the probability that the compound or constituent was positive as well as the probability that the compound or constituent was negative.

Table 17 provides a summary of the three models. The sentiment value for the compound is a successful predictor of lexical decision as well as of naming. However, the sentiment values for the individual constituents did not predict response times in either task, even when the sentiment values for the entire compound were not entered into the model. Both the probability of being negative and the probability of being positive have positive coefficients (i.e., lower value are associated with longer times). This outcome suggests that the higher the probability of compound to be either positive or negative aids processing. This could be an arousal effect (i.e., the more arousing the word is, the faster participants respond). The idea is that emotional content, whether positive or negative, tends to be arousing, as compared to completely neutral stimuli. These results suggest that the sentiment classification may be a useful measure in psycholinguistic research. Furthermore, it appears that knowledge about sentiment plays a role only at the whole-word level, or that the sentiments associated with the constituents are quickly suppressed during processing.

**Table 17:** Standardized regression coefficients with standard errors (in parentheses) from models using frequency, stimulus length (in letters), and sentiment to predict English Lexicon Project (ELP) lexical decision (LD) times, British Lexicon Project (BLP) lexical decision times, and ELP naming times

	ELP LD	BLP LD	BLP LD	ELP Naming
SUBTLEX frequency	– 0.473^***^	– 0.455^***^		– 0.401^***^
	(0.00162)	(0.00149)		(0.00136)
BNC frequency			– 0.464^***^	
			(0.00132)	
stimlen	0.226^***^	– 0.012	0.042^*^	0.264^***^
	(0.000647)	(0.000773)	(0.000743)	(0.000544)
sentimentprobneg_stim	0.090^***^	0.126^***^	0.115^***^	0.065^**^
	(0.0219)	(0.0212)	(0.0221)	(0.0184)
sentimentprobneg_c1	– 0.010	– 0.010	0.004	– 0.041
	(0.0112)	(0.0107)	(0.0104)	(0.00942)
sentimentprobneg_c2	0.016	0.012	– 0.003	0.008
	(0.0125)	(0.0120)	(0.0114)	(0.0105)
sentimentprobpos_stim	0.073^***^	0.083^**^	0.052^*^	0.050^*^
	(0.0215)	(0.0213)	(0.0225)	(0.0181)
sentimentprobpos_c1	– 0.025	– 0.002	– 0.017	– 0.015
	(0.0114)	(0.0107)	(0.0104)	(0.00961)
sentimentprobpos_c2	0.025	0.019	0.012	0.023
	(0.0131)	(0.0124)	(0.0118)	(0.0110)
* N*	2,593	2,002	2,396	2,594
adj. R-sq	0.320	0.231	0.244	0.267
AIC	– 8,187.5	– 7,089.5	– 8,242.7	– 9,082.9

Standardized beta coefficients; standard errors are in parentheses. ^*^p < .05, ^**^p < .01, ^***^p < .001.

We conducted a similar set of analyses, using Warriner et al.’s (2013) valence ratings, which range from 1 (*negative/unhappy*) to 9 (*positive/happy*). Table 18 provides a summary of the three models. As with the analyses using sentiment, the valence of the compound was a successful predictor of both BLP and ELP lexical decision times. Compounds with negative valences (i.e., lower ratings) were associated with slower lexical decision times. This finding is consistent with Kuperman et al. (2014), who also found that negative words (e.g., *coffin*) take longer to process than neutral (e.g., *cotton*) and positive (e.g., *kitten*) words. However, unlike in the sentiment analysis, valence was not a successful predictor of naming latencies. In terms of the valence of the constituents, valence was not predictive except for the valence of the second constituent for the BLP lexical decisions times when compound frequency was estimated by SUBTLEX-US. The valence of the second constituent was not predictive when the compound frequency was estimated by BNC frequency. The null effects of the constituent valences differ from the results of Kuperman (2013), who found that the valence of constituents affected ELP lexical decision times. A detailed investigation of this difference is beyond the scope of this article, but two potential explanations are that the item sets differ (Kuperman, 2013, used 557 items), and also that Kuperman used residualization to remove collinearity between the valences of the two constituents and the compound (see Wurm & Fisicaro, 2014, for a discussion of why residualization might not be optimal).

**Table 18:** Standardized regression coefficients with standard errors (in parentheses) from models using frequency, stimulus length (in letters), and valence to predict English Lexicon Project (ELP) lexical decision (LD) times, British Lexicon Project (BLP) lexical decision times, and ELP naming times

	ELP LD	BLP LD	BLP LD	ELP Naming
SUBTLEX frequency	– 0.302^***^ (0.00277)	– 0.417^***^(0.00205)		– 0.290^***^ (0.00215)
BNC frequency			– 0.534^***^ (0.00159)	
stimlen	0.199^***^ (0.000965)	– 0.032 (0.00105)	0.009 (0.000945)	0.248^***^ (0.000750)
valence_stim	– 0.165^***^ (0.00144)	– 0.218^***^ (0.00126)	– 0.151^***^ (0.00117)	– 0.049 (0.00112)
valence_c1	– 0.054 (0.00136)	– 0.022 (0.00116)	– 0.015 (0.00108)	– 0.055 (0.00106)
valence_c2	– 0.003 (0.00151)	0.087^**^ (0.00137)	0.046 (0.00127)	– 0.004 (0.00117)
* N*	1,076	950	950	1,076
adj. R-sq	0.187	0.223	0.334	0.168
AIC	– 3,605.7	– 3,567.7	– 3,716.5	– 4,148.0

Standardized beta coefficients; standard errors are in parentheses. ^*^p < .05, ^**^p < .01, ^***^p < .001.

## General discussion

LADEC fills an important gap in the field, due to its size and inclusion of measures that are unique to compound words. The database contains over 8,000 compounds along with information covering a range of levels, from orthographic to morphological to semantic. In addition, the database includes semantic transparency ratings (collected in a lab setting) from a total of almost 1,800 participants. These semantic transparency ratings assess transparency from both compound-based and constituent-based perspectives. By collecting ratings that target both of the individual constituents’ transparency as well as the compound-based transparency, we were able to get a more fine-grained glimpse into how the various aspects of semantic transparency are interrelated, as well as into how they work together to influence compound processing. The complete list of variables included in the database can be found in the Appendix.

To examine the viability of some of the variables and provide examples of some of the types of questions that can be addressed using LADEC, we conducted several sets of analyses focusing on different aspects of the information contained in the database. The analyses concerning semantic transparency established that our ratings of semantic transparency correlate with previous measures reported in the literature (Juhasz et al., 2015; Kim et al., 2018), but also are more consistent predictors across three sets of response time data (ELP naming, ELP lexical decision, and BLP lexical decision) than the previous measures. For example, whereas the semantic transparency ratings in Juhasz et al. (2015) predicted only BLP lexical decision times, our meaning predictability and meaning retention ratings of the first constituent predicted all three types of response times, and meaning retention ratings of the second constituent predicted both types of lexical decision times for the full set, and ELP lexical decision times for the reduced set. The influence of the meaning retention ratings of the second constituent on naming emerged only when the compound-based, meaning predictability rating was included in the model, and only for the full set of items.

We also found evidence that processing is sensitive to other sources of influence, such as orthographic and emotional information. The analyses using bigram frequency indicated that compound processing is sensitive to this orthographic information and that high-frequency bigrams at the morpheme boundary slow processing. This slowdown is consistent with claims that bigram frequency provides a cue for morphemic parsing (Seidenberg, 1987), in that higher frequency makes it more difficult to identify the boundaries of the constituents (or, alternatively, that low-frequency bigrams are taken as evidence of a boundary, and hence encourage parsing at that point).

The analyses with sentiment indicate that this measure, which was generated via an autoclassifier, might be a useful measure for further research examining the impact of emotion on lexical processing. Indeed, as was demonstrated by Kuperman (2013), measures such as valence appear to impact even the early stages of complex-word processing. Having sentiment values available for a large set of items (as in LADEC) can aid in the further exploration of this issue.

Another useful aspect of our results is that they indicate that it is useful for researchers to separately consider the contribution of each constituent, because their contributions may not be symmetric. For example, we found that although the meaning retention ratings for both constituents were associated with the compound-based meaning predictability measure (e.g., how predictable *schoolteacher* is from *school* and *teacher*), the meaning retention ratings for the first constituent were more strongly associated with the compound-based measure than were the meaning retention ratings for the second constituent. It is possible that this occurs because the modifier (the first constituent in English compounds) has a larger impact on specifying the way in which the category denoted by the compound differs from the head category, particularly for relatively transparent compounds. For example, the first constituent creates the variation between *bluebird*, *seabird*, and *hummingbird*. In addition, as is shown in Fig. 1, the distribution of transparencies for the second constituent is much more peaked (and higher) than the distribution for the first constituent, indicating a somewhat stronger bias toward transparency in the second constituent overall, and far more so than for the compound as a whole. Thus, there seems to be a bias toward the second constituent’s transparency, such that the transparency of the whole compound then depends more on the transparency of the first constituent.

Similarly, the analyses with family size indicate that the influences of first- and second-constituent family size are not symmetric. The family size of the first constituent, but not of the second, plays a role in predicting lexical decision latencies. At first glance, this finding might seem counterintuitive, because most theories of complex-word processing posit that the head constituent plays a more important role in the representation of the compound (Libben et al., 2003). However, past research has also indicated that, in terms of processing, the first constituent in English (i.e., the modifier concept) tends to play a more influential role (Gagné, 2002; Gagné & Spalding, 2009). Thus, our present finding concerning the impact of constituent family size fits well with the latter research—namely, it suggests that the more information participants have about how a modifier concept tends to be used, the more readily they can process the compound. This ease could arise because the constituent is more easily accessed and assigned its role in the morphological structure. Indeed, Gagné et al. (2009) reported that participants took less time to process a noun phrase when the previous trial had used a constituent in the same position (i.e., when both used the same word as the first constituent) than when the previous trial had used the constituent in a different position (i.e., when the constituent was used as the second constituent in the previous trial but as the first constituent in the current trial). Alternatively, the ease of processing due to a larger first-constituent family could be due to that family facilitating the ease of selecting a semantic relation that links the two constituents within the conceptual representation that corresponds to the lexical representation. Ease of selecting a semantic relation has also been shown to influence the ease of processing for both novel and familiar (lexicalized) compounds (for an overview, see Gagné & Spalding, 2014).

Overall, the various sets of analyses in this article support the idea that compound processing is multifaceted and draws on information ranging from orthographic information (e.g., bigram frequency), to morphological information (e.g., family size), to semantic/conceptual information (e.g., semantic transparency and sentiment) about the compound and its constituents. In addition, these analyses show some of the important asymmetries between the effects of the individual constituents in compound processing.

In conclusion, LADEC can be used in a variety of ways. As we have shown above, it will allow researchers to run virtual experiments on a dataset to check new hypotheses, or to confirm the generalizability of existing findings. It can also be used to select materials for future experiments and will allow researchers to search for items with particular behavioral or stimulus characteristics (e.g., compounds with transparency ratings within a certain range). The size of the database provides greater options for matching and selecting items on a broader range of criteria. In addition, the database is useful for finding control stimuli when investigating both monomorphemic words and derived words. Importantly, the database can also be a useful test set for examining the accuracy of autoclassification and morphological-parsing systems. For example, Tucker et al. (2019) noted that the 1,200 noun–noun compounds incorporated in the Englex English morpheme sets (Antworth, 1994) were mostly analyzed as single morphemes by the PC-Kimmo two-level morphological parser. Items from LADEC could be used as input to evaluate the accuracy of such parsers. Similarly, the rating data were collected in a lab setting, and thus could also serve as a useful tool for researchers wishing to evaluate differences (if any) between crowd-sourced data collection (from, e.g., mTurk) and data collected in a one-on-one setting. Thus, we believe that LADEC will, in many respects, facilitate research on the processing of compound words, in particular, and of morphologically complex words, in general.

### Availability and Open Practices Statement

The LADEC dataset is available for public download at https://era.library.ualberta.ca (search term: LADEC), formatted as text (.csv) or as a Stata datafile. The do-file for producing the analyses reported in this article is also available at that location. When using variables that were obtained from other sources, please cite the original source. None of the experiments were preregistered.

## Appendix

**Table 19.** List of variables in LADEC

Variable Name	Variable Description
id_master	id
c1	first constituent
c2	second constituent
stim	compound
obs	observation number by compound
obsc1	observation number by first constituent
obsc2	observation number by second constituent
stimlen	length of compound
c1len	length of first constituent
c2len	length of second constituent
nparses	number of parses per compound
correctParse	correct parse? (1 = *yes*, 0 = *no*)
ratingcmp	Predictability rating
ratingC1	Meaning retention rating for first constituent
ratingC2	Meaning retention rating for second constituent
isPlural	is plural? (1 = *yes*, 0 = *no*)
nc1_cmp	first constituent, family size based on correctly parsed compounds
nc2_cmp	second constituent, family size based on correctly parsed compounds
nc1_cmpnoplural	first constituent, family size based on correctly parsed compounds, no plurals
nc2_cmpnoplural	second constituent, family size based on correctly parsed compounds, no plurals
sentiment_stim	sentiment stim (Mathematica classifier)
sentiment_c1	sentiment first constituent (Mathematica classifier)
sentiment_c2	sentiment second constituent (Mathematica classifier)
sentimentprobpos_stim	probability of positive sentiment, stimulus (Mathematica classifier)
sentimentprobpos_c1	probability of positive sentiment, first constituent (Mathematica classifier)
sentimentprobpos_c2	probability of positive sentiment, second constituent (Mathematica classifier)
sentimentprobneg_stim	probability of negative sentiment, stimulus (Mathematica classifier)
sentimentprobneg_c1	probability of negative sentiment, first constituent (Mathematica classifier)
sentimentprobneg_c2	probability of negative sentiment, second constituent (Mathematica classifier)
sentimentratioposneg_stim	ratio of probPos/probNeg, stimulus (Mathematica sentiment classifier)
sentimentratioposneg_c1	ratio of probPos/probNeg, first constituent (Mathematica sentiment classifier)
sentimentratioposneg_c2	ratio of probPos/probNeg, second constituent (Mathematica sentiment classifier)
profanity_stim	is stimulus profane? (Mathematica classifier)
profanity_c1	is first constituent profane? (Mathematica classifier)
profanity_c2	is second constituent profane? (Mathematica classifier)
isCommonstim	is stimulus in Mathematica_CommonList?
isCommonC1	Is first constituent in Mathematica's list of common words? (1 = *yes*, 0 = *no*)
isCommonC2	Is second constituent in Mathematica's list of common words? (1 = *yes*, 0 = *no*)
bg_boundary	bigram at c1c2 boundary
bgJonesMewhort	bigram frequency from Jones & Mewhort (2004)
bgSUBTLEX	bigram frequency from SUBTLEX-US
bgFacebook	bigram frequency from Facebook
inSUBTLEX	compound in SUBTLEX-US? (1 = *yes*, 0 = *no*)
inBLP	compound in BLP? (1 = *yes*, 0 = *no*)
inELP	compound in ELP? (1 = *yes*, 0 = *no*)
inJuhaszLaiWoodcock	compound in Juhasz, Lai, & Woodcock (2015)? (1 = *yes*, 0 = *no*)
c1_inELP	first constituent in ELP? (1 = *yes*, 0 = *no*)
c1_inBrysbaert	first constituent in Brysbaert, Warriner, & Kuperman (2014)? (1 = *yes*, 0 = *no*)
c1_inWordnet	first constituent in Wordnet? (1 = *yes*, 0 = *no*)
c1_inMMA	first constituent in Mathematica? (1 = *yes*, 0 = *no*)
c2_inELP	second constituent in ELP? (1 = *yes*, 0 = *no*)
c2_inBrysbaert	second constituent in Brysbaert, Warriner, & Kuperman (2014)? (1 = *yes*, 0 = *no*)
c2_inWordnet	second constituent in Wordnet? (1 = *yes*, 0 = *no*)
c2_inMMA	second constituent in Mathematica? (1 = *yes*, 0 = *no*)
LSAc1c2	LSA between first constituent and second constituent
LSAc1stim	LSA between first constituent and compound
LSAc2stim	LSA between second constituent and compound
stim_SLlg10wf	SUBTLEX-US log word frequency for compound
C1_SLlg10wf	SUBTLEX-US log word frequency for first constituent
C2_SLlg10wf	SUBTLEX-US log word frequency for second constituent
BLPbncfrequency	BNC word frequency of the compound from the BLP
BLPbncfrequencymillion	BNC word frequency per million of the compound from the BLP
c1_BLPbncfrequency	BNC word frequency of the first constituent from the BLP
c1_BLPbncfrequencymillion	BNC word frequency per million of the first constituent from the BLP
c2_BLPbncfrequency	BNC word frequency of the second constituent from the BLP
c2_BLPbncfrequencymillion	BNC word frequency per million of the second constituent from the BLP
BLPrt	BLP lexical decision times
elp_ld_rt	ELP lexical decision times
elp_naming_mean_rt	ELP naming times
c1c2_snautCos	first and second constituent cosine distance SNAUT
c1stim_snautCos	first constituent and compound cosine distance SNAUT
c2stim_snautCos	second constituent and compound cosine distance SNAUT
fbusfreq	frequency per billion words in the American corpus
fbukfreq	frequency per billion words in the British corpus
valence_stim	valence of compound in Warriner, Kuperman, & Brysbaert (2013)
valence_c1	valence of first constituent in Warriner, Kuperman, & Brysbaert (2013)
valence_c2	valence of second constituent in Warriner, Kuperman, & Brysbaert (2013)
concreteness_stim	concreteness of compound in Brysbaert, Warriner, & Kuperman (2014)
concreteness_c1	concreteness of first constituent in Brysbaert, Warriner, & Kuperman (2014)
concreteness_c2	concreteness of second constituent in Brysbaert, Warriner, & Kuperman (2014)
Juhasz_tran (2015_trans)	Transparency rating from Juhasz, Lai, & Woodcock (2015)
st_c1_mean (2018_ratingC1)	Rating for first constituent from Kim et al. (2018)
st_c2_mean (2018_ratingC2)	Rating for first constituent from Kim et al. (2018)
